# tRF-Gln-CTG-026 ameliorates liver injury by alleviating global protein synthesis

**DOI:** 10.1038/s41392-023-01351-5

**Published:** 2023-04-03

**Authors:** Sunyang Ying, Pengcheng Li, Jiaqiang Wang, Kaiqiong Chen, Yu Zou, Moyu Dai, Kai Xu, Guihai Feng, Changjian Zhang, Haiping Jiang, Wei Li, Ying Zhang, Qi Zhou

**Affiliations:** 1 State Key Laboratory of Stem Cell and Reproductive Biology, Institute of Zoology, Chinese Academy of Sciences, Beijing, 100101 China; 2 Institute for Stem Cell and Regenerative Medicine, Chinese Academy of Sciences, Beijing, 100101 China; 3 University of Chinese Academy of Sciences, Beijing, 100049 China; 4 College of Life Science, Northeast Agricultural University of China, Harbin, 150030 China; 5 Beijing Institute for Stem Cell and Regenerative Medicine, Beijing, 100101 China; 6 Central Laboratory of the Sixth Medical Center of PLA General Hospital, Beijing, 100048 China

**Keywords:** Non-coding RNAs, Cell biology

## Abstract

tsRNAs (tRNA-derived small RNAs), as products of the stress response, exert considerable influence on stress response and injury regulation. However, it remains largely unclear whether tsRNAs can ameliorate liver injury. Here, we demonstrate the roles of tsRNAs in alleviating liver injury by utilizing the loss of *NSun2* (NOP2/Sun domain family, member 2) as a tsRNAs-generating model. Mechanistically, the loss of *NSun2* reduces methyluridine-U5 (m^5^U) and cytosine-C5 (m^5^C) of tRNAs, followed by the production of various tsRNAs, especially Class I tsRNAs (tRF-1s). Through further screening, we show that tRF-Gln-CTG-026 (tG026), the optimal tRF-1, ameliorates liver injury by repressing global protein synthesis through the weakened association between TSR1 (pre-rRNA-processing protein TSR1 homolog) and pre-40S ribosome. This study indicates the potential of tsRNA-reduced global protein synthesis in liver injury and repair, suggesting a potential therapeutic strategy for liver injury.

## Introduction

The liver performs various biological functions to maintain homeostasis, e.g., glycogen storage, nutrient metabolism, drug detoxification, bile secretion, and protein synthesis. When liver injury occurs, hepatocytes experience tremendous damage stress, which can cause various conditions, including hemorrhagic necrosis, hepatocellular apoptosis, hydropic degeneration, hepatic steatosis, and liver inflammation.^1,2^ The mechanisms of liver injury are complex and diverse, but converge to two aspects: immune and biochemical.^3^ Liver injury is an area of concern as it can initiate a series of abnormal apoptosis programs. Liver injury resulting from acute liver failure (ALF), cirrhosis, cancer, or other causes is responsible for numerous deaths worldwide. Liver transplantation is almost the only treatment to promote survival. However, the side effects and shortage of donor livers restrict this treatment. The liver is an organ into which small RNAs can be efficiently delivered; therefore, small RNA therapeutics may illuminate a new avenue in liver injury. Current small RNA therapeutics mainly rely on siRNAs. However, because of their immunogenicity, toxicity, and degradation, the development of novel small RNA therapeutics that resolve these issues is imperative.

tsRNAs (tRNA-derived small RNAs) are not random by-products from degrading tRNAs, but instead are accurately controlled non-coding small RNAs. tsRNAs are generally produced by recruiting ribonucleases, such as Dicer, ELAC2/RNase Z, and Angiogenin, to cleave tRNA.^4,5^ Based on the cleavage positions of mature or precursor tRNA, tsRNAs can be typically categorized into tRF-1s, tRF-3s, tRF-5s, and tRNA halves (tiRNAs).^6^ Accumulated evidence indicates that tsRNAs are involved in stress responses and have a broad association with injury.^7,8^ Under stress, tsRNAs can regulate ribosome biogenesis to control protein translation, promote stress granule (SG) assembly, and mediate the process of apoptosis.^5,6,9–11^ A recent study reported the proliferative effect of tsRNAs under damage stress.^12^ Despite their vital roles in regulating stress response and injury, owing to the lack of therapeutically focused research, tsRNAs have not been developed as small therapeutic RNAs to alleviate diseases.

To elucidate the roles of tsRNAs in the treatments of liver injury, an effective model to generate tsRNAs is necessary. It was reported that *NSun2* (NOP2/Sun domain family, member 2), a tRNA methyltransferase, is required to maintain tRNA modification, stabilize mature tRNA structures, and protect tRNAs from rapid degradation. *NSun2* depletion causes robust hypomethylation of the tRNA cytosine-C5 (m^5^C), followed by recruitment of nucleases to cut tRNAs, resulting in the accumulation of tsRNAs.^13–15^ Therefore, *NSun2* knockout (NS-KO) is a valuable model to explore the following question: as the by-products of the stress response, do tsRNAs participate in repairing liver injury? It is widely reported that tsRNAs regulate protein translation and are involved in ribosomal regulation.^16,17^ Moreover, the inhibition of global protein synthesis (GPS) can reduce protein misfolding and unfolding to promote survival.^18,19^ Hence, we speculate that tsRNAs can reduce GPS to alleviate liver injury.

Here, we establish the relationship between tsRNAs and liver injury and demonstrate that NS-KO changes tRNA posttranscriptional modifications and causes the generation of Class I tsRNAs (tRF-1s). Through screening, we also provide further evidence that tRF-Gln-CTG-026 (tG026), among NS-KO-derived tRF-1s, significantly ameliorates liver injury by suppressing GPS. Collectively, the results show that tG026 is a prospective therapeutic strategy to relieve liver injury-associated diseases.

## Results

### Establishing loss-of-*NSun2* as a tsRNA-generating model to alleviate injury in vitro

**Fig. 1:**
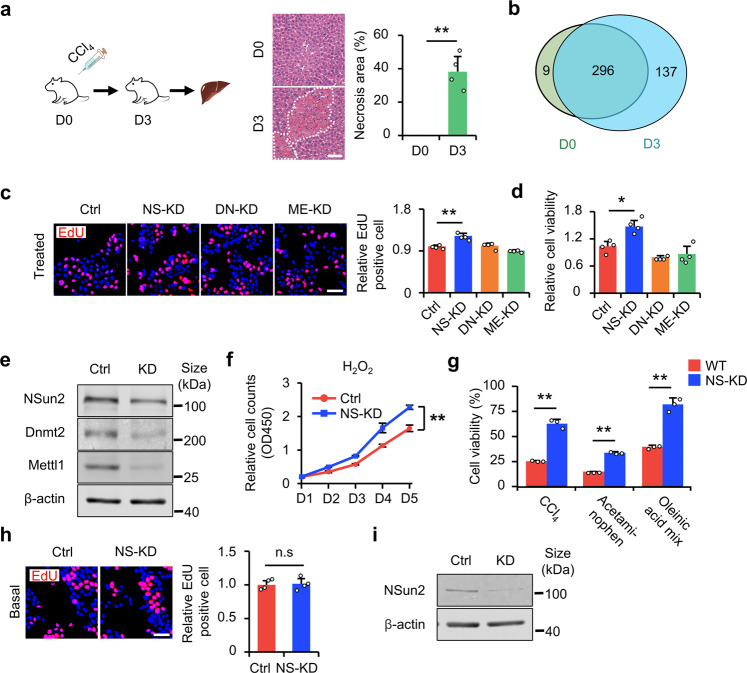
NS-KD alleviates cell injury. **a** CCl_4_-induced liver injury. Left panel: schematic of the construction of liver injury mice models. Liver injury was induced by CCl_4_ injection. The lower part indicates the time, i.e., D0: intraperitoneally (i.p.) injection of CCl_4_ or vehicle on day 0. D3: assessing the extent of liver injury on day 3. Right panel: H&E staining showing hepatic necrosis following injection of vehicle (D0) or CCl_4_ (D3). Dotted lines indicate the necrotic or inflammatory area, and the percentage of the necrotic or inflammatory area is quantified. Scale bar, 50 μm; *n* = 4 mice. **b** Venn diagram showing the numbers of tsRNAs in the presence or absence of CCl_4_ stress. The color represents samples with or without CCl_4_ stress. The numeral is the number of tsRNAs in the corresponding area. The data are acquired by tsRNA-seq, and each group comprised two duplicate samples. **c** The effect of four tRNA modification enzymes on HL-7702 proliferation under H_2_O_2_ stress. Treated: H_2_O_2_. Left panel: representative images of EdU staining are shown. Right panel: statistical results. Scale bar, 100 μm. EdU-positive cells were analyzed as data relative to the control *n* = 4 replicates. **d** Relative HL-7702 viability (CCK-8 assay) was assessed by detecting the OD450 value. *n* = 4 replicates. **e** NSun2 expression was measured to verify the knockdown effect of NSun2 by western blotting in (**c**) and (**d**). **f** Detection of HL-7702 proliferation using CCK-8 assay under H_2_O_2_ stress at the indicated time points (day 1, day 2, day 3, day 4, day 5), **P < 0.01, versus control siRNA at day 5. **g** Detection of transfected HL-7702 cell survival after stimulation with other chemicals. HL-7702 viability was assessed by the CCK-8 assay. *n* = 3 replicates. **h** Detection of transfected HL-7702 cell proliferation in the absence of stress damage. Basal: no H_2_O_2_. Left panel: representative images of EdU staining. Right panel: statistical results. Scale bar, 100 μm. EdU-positive cells were analyzed relative to the control. *n* = 4 replicates. **i** NSun2 expression was measured to verify the knockdown effect of NSun2 by western blotting for (**f**–**h**) and Supplementary Fig. S1j. *NSun2* siRNA: NS-KD; Ctrl siRNA: Ctrl. All data are expressed as the mean ± SD. *P < 0.05, **P < 0.01, *n.s*. no significance, Student’s *t*-test

We first induced liver injury in mice to verify whether tsRNAs were involved in liver injury and repair (Fig. 1a). We found that the number of tsRNAs increased in mice with liver injury (Fig. 1b), demonstrating that the generation of tsRNAs is related to liver injury. To further study the relationship between tsRNAs and liver injury, it is necessary to establish a tsRNAs-generating model. Before establishing model, we first define the standard of high and low damage stress in cell experiments in vitro. We performed H_2_O_2_ dose gradient experiments in both HL-7702 and AML12 cells. The EdU incorporation results showed that under >10 μM H_2_O_2_ stress, cell proliferation was significantly inhibited in HL-7702 and AML12 cells (Supplementary Fig. S1a–c). With the increase of dose, no proliferative cells in HL-7702 cells under 500 μM H_2_O_2_ stress (Supplementary Fig. S1b) and AML12 cells under 60 μM H_2_O_2_ stress because cells die (Supplementary Fig. S1c). Hence, we define 10 μM H_2_O_2_ stress as low H_2_O_2_ stress or proliferation stress, which cannot trigger cells death, but inhibit cell growth. On the other hand, CCK-8 assay suggests that under 500 μM H_2_O_2_ stress, most of HL-7702 cells die, and under 100 μM H_2_O_2_ stress most of AML12 cells die. For the concentration less than 500 μM for HL-7702 and 100 μM for AML12, cells cannot significantly die. For the concentration higher than 500 μM for HL-7702 and 100 μM for AML12, cells will all die (Supplementary Fig. S1d, e). Therefore, we define 500 μM H_2_O_2_ stress for HL-7702 and 100 μM H_2_O_2_ stress for AML12 as high H_2_O_2_ stress, lethal stress, or survival stress. From screening of three tRNA modification enzymes (NSun2, Mettl1, and Dnmt2) in the HL-7702 cell line, under low damage stress, *NSun2* knockdown (NS-KD) was shown to result in increased proliferation compared with ME-KD (*Mettl1* knockdown) and DN-KD (*Dnmt2* knockdown) (Fig. 1c, e); in addition, under high (lethal) stress, *NSun2* knockdown increased cell survival (Fig. 1d, e). We also confirmed the results in the AML12 cell line (Supplementary Fig. S1f, g, i). To further explore the roles of NS-KD in cell injury, we measured cell proliferation for 5 days continuously under low H_2_O_2_ stress using a growth curve experiment. Our results showed that NS-KD increased cellular proliferation under the H_2_O_2_ treatment (Fig. 1f). In contrast, we applied different high-intensity stress inducers (CCl_4_, acetaminophen, and oleinic acid mixture) and measured cell survival. The results show that NS-KD improved cell survival under stress damage (Fig. 1g, i). As expected, NS-KD functions only under stress. Under basal conditions, NS-KD does not affect cell proliferation (Fig. 1h, i and Supplementary Fig. S1h, i). Furthermore, to determine the effect of *NSun2* loss on cell migration, we performed a cell migration assay using HL-7702 cells and found that the migration of HL-7702 remained unchanged after NS-KD (Supplementary Fig. S1j). Collectively, these results suggest that liver injury is related to tsRNA generation, and that NS-KD promoted cell proliferation and survival but did not change the cell migration under stress in vitro.

### NS-KO ameliorates liver injury in vivo

**Fig. 2:**
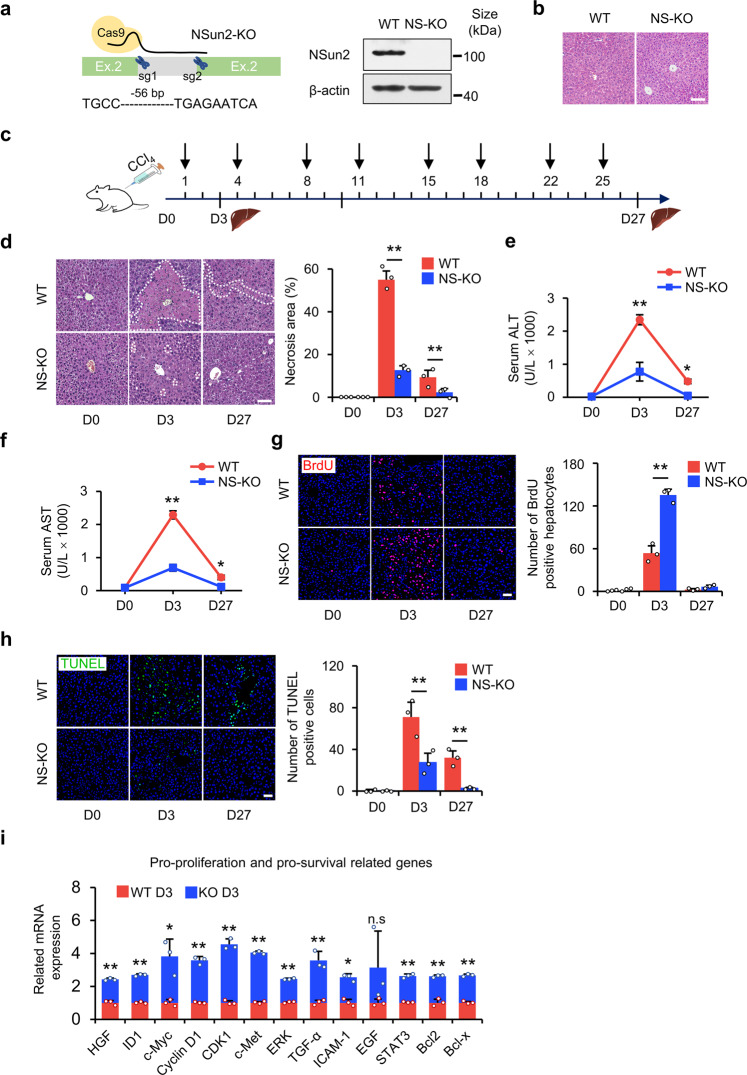
NS-KO improves liver necrosis, regeneration, and survival in vivo. **a** Generation of NS-KO mice. Left panel showing a schematic representation of NS-KO mice, *NSun2* genomic locus, and *NSun2* deleted locus. The targeting sgRNA was designed to knock out 56 bp DNA fragments in exon 2 of the *NSun2* gene. The right panel is genotyping analysis of WT and NS-KO mice confirmed by western blotting. **b** H&E staining showing mouse liver in WT and NS-KO mice not subjected to stress. Scale bar, 50 μm; *n* = 3 mice. **c** Schematic of the construction of liver injury mice models. Liver injury was induced by repeated injections of CCl_4_. The part above the arrow line of time represents the injection time, and the part under the arrow line represents the liver-obtained time. D0: no injury; D3: day 3 after injury; D27: day 27 after injury. **d** H&E staining showing hepatic necrosis at different time points after CCl_4_ injection. Dotted lines indicate the necrotic or inflammatory area and the percentage of the necrotic or inflammatory areas is quantified. Scale bar, 50 μm; *n* = 3 mice. **e**, **f** Mouse serum ALT (**e**) and AST (**f**) concentrations were assessed to determine the degree of liver injury after repeated CCl_4_ injection, *n* = 4 mice. **g** Hepatocytes proliferation in liver sections. Three hours before the mice were euthanized, 5-bromodeoxyuridine (BrdU) incorporation was performed for WT and NS-KO mice treated with CCl_4_ and BrdU-positive hepatocytes were quantified. Scale bars, 200 μm; *n* = 3 mice. **h** TdT-mediated dUTP nick end labeling (TUNEL) assay on liver sections of WT mice or NS-KO mice treated with CCl_4_. Green cells indicate TUNEL-positive (apoptotic) cells. Nuclei were stained with DAPI, and TUNEL-positive nuclei were quantified. Scale bars, 200 μm; *n* = 3 mice. **i** Proliferation- and survival-related gene expression was quantified by qRT-PCR after liver injury. *n* = 3 mice. For data in this figure, representative images are shown. All data are presented as the mean ± SD. *P < 0.05, **P < 0.01, Student’s *t*-test

As mentioned above, the loss of *NSun2* is an appropriate model to study tsRNAs^15^ and can improve cell proliferation and survival under stress. Therefore, we generated NS-KO mice via CRISPR/Cas9-mediated 56 bp deletion in exon 2 of the *NSun2* gene (Fig. 2a and Supplementary Fig. S2a), and, through immunohistochemical staining, demonstrated that NSun2 was localized in the nucleus of both parenchymal and nonparenchymal cells under non-stress conditions (Supplementary Fig. S2b). Functionally, we first verified whether the *NSun2* deletion mutant responded to the stress of liver injury. In the absence of stress damage, NS-KO did not cause abnormalities in liver morphology and pathophysiology (Fig. 2b), indicating that neither NS-KO nor the consequently produced tsRNAs functioned in the absence of stress. After modeling short- or long-term liver injury by intraperitoneal injection (i.p.) of CCl_4_ (Fig. 2c), we demonstrated that NS-KO responded to stress damage and attenuated liver injury. On day 3 after a single CCl_4_ injection, necrosis and inflammation were observed in the liver; on day 27, fibrosis was observed. The degree of necrosis was lower in NS-KO mice than in wild-type (WT) mice (Fig. 2d). Next, we examined the blood biochemical indices after single or repeated injection of CCl_4_. Alanine aminotransferase (ALT), aspartate aminotransferase (AST), and alkaline phosphatase (ALP) concentrations reached their maximum values on day 3 after a single CCl_4_ injection, indicating considerably severe hepatocyte damage, and afterward decreased to relatively normal levels on day 27 of repeated CCl_4_ injection. All three indicators in NS-KO mice were lower than those observed in WT mice, implying minor liver damage in NS-KO mice (Fig. 2e, f and Supplementary Fig. S2c). Similarly, long-term injury generates fibrosis. The extent of fibrosis in NS-KO mice was milder than that in WT mice (Supplementary Fig. S2d). To investigate how the loss of *NSun2* ameliorates liver injury, we measured hepatocyte proliferation through BrdU incorporation and Ki67 staining assays. Compared with WT mice, we found that the number of proliferating hepatocytes in NS-KO mice was significantly higher on day 3 of liver injury (Fig. 2g and Supplementary Fig. S2e). We also examined how *NSun2* loss affects cell death using the TdT-mediated dUTP nick end labeling (TUNEL) assay. The results suggested that the number of apoptotic hepatocytes peaked on day 3 of liver injury and then progressively declined. Fewer apoptotic hepatocytes were detected in NS-KO mice than in WT mice, implying that the loss of *NSun2* resisted cell apoptosis (Fig. 2h). The expression of pro-proliferation, pro-survival, and anti-inflammation-related genes was increased under stress, whereas the expression of anti-proliferation, anti-survival, and pro-inflammation-related was suppressed (Fig. 2i and Supplementary Fig. S2f, g). In sum, we observed milder liver damage in NS-KO mice than WT mice in both short-term and long-term liver injury, suggesting that *NSun2* loss attenuates liver injury in vivo.

### NS-KO-derived small RNAs improve proliferation and survival under stress

**Fig. 3:**
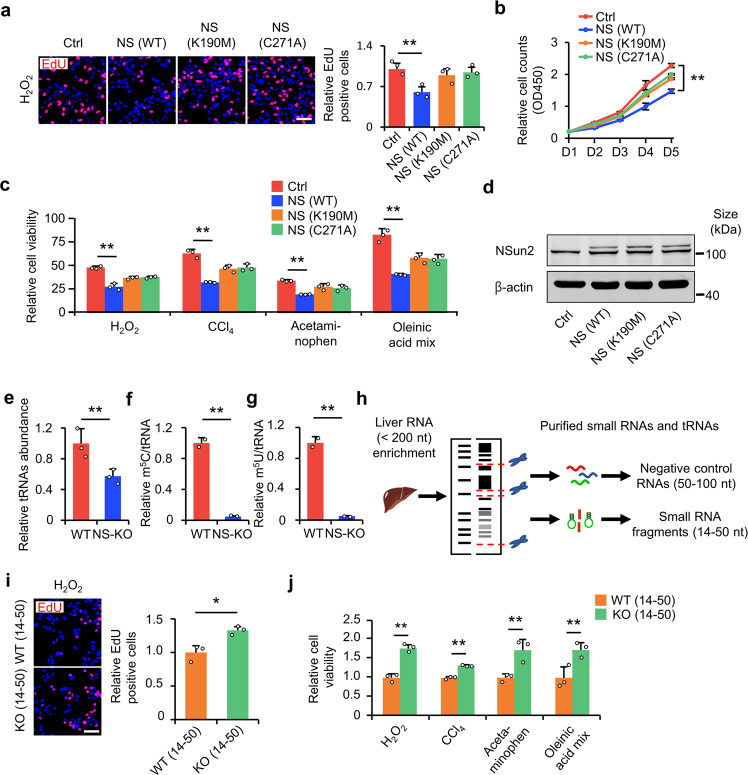
NS-KO-derived tsRNAs alleviate liver injury. **a** *NSun2*-knockdown HL-7702 cells were rescued with wild-type *NSun2* (NS), two catalytically dead mutants (K190M, C271A), or the empty vector. Relative levels of EdU-positive cells were determined. *n* = 3 replicates. **b** Cell proliferation assay (CCK-8) of *NSun2*-knockdown HL-7702 rescued by enzymatic-dead *NSun2*. Detection of OD450 at the indicated times (day 1, day 2, day 3, day 4, day 5) under H_2_O_2_ stress, **P < 0.01, versus NS-KD at day 5. **c** Cell viability detection of *NSun2*-knockdown HL-7702 rescued by enzymatic-dead *NSun2*. Relative cell viability was assessed by CCK-8 assay. *n* = 3 replicates. The data in Fig. 3c and Fig. 1g are from the same batch of experiments. **d** *NSun2* expression was measured to verify the rescue effect of *NSun2* by western blotting in (**a**–**c**). **e** tRNA abundance in WT and NS-KO mice. Data indicate the tRNA abundance relative to WT, *n* = 3 mice. **f**, **g** Liquid chromatography-mass spectrometry showing the differences of tRNA m^5^U (**f**) and m^5^C (**g**) modifications in WT and NS-KO mice. Data are shown as a relative value (tRNA modification/total tRNA), *n* = 2 mice. **h** Schematic for isolating small liver RNAs. **i** EdU assay showing the proliferation of isolated fragments. EdU-positive cells were analyzed as data relative to WT. Scale bar, 100 μm. *n* = 3 replicates. **j** CCK-8 assay showing the survival of isolated fragments. Relative cell viability (CCK-8 assay) of 14–50 nt RNAs transfection was assessed by detecting the OD450 value. *n* = 3 replicates. For all data in this figure representative images are shown. All data are presented as the mean ± SD. *P < 0.05, **P < 0.01, Student’s *t*-test

We hypothesized that NS-KO prevents liver injury through NS-KO-derived small RNAs. To test this hypothesis, we first knocked down *NSun2* in the HL-7702 cell line, transfected cells with mutant *NSun2* (K190M or C271A), which has no enzymatic activity, into the HL-7702 cell line, and subjected the cells to cytotoxic stimuli. The EdU incorporation and cell proliferation assays showed that only WT *NSun2* inhibited cell proliferative promotion caused by *NSun2* knockdown (Fig. 3a, b, d) instead of *NSun2* mutant (K190M or C271A). Similarly, only WT *NSun2* reversed the increase in cell viability induced by NS-KD; the *NSun2* mutants did not (Fig. 3c, d). These results demonstrated that the loss of *NSun2* promotes cell proliferation and survival under stress mainly through the loss of tRNA methyltransferase activity. When NSun2 loses its methyltransferase activity, tRNA modifications are inevitably reduced. To confirm this hypothesis, we measured multiple tRNA modifications (41 types of modifications) in the liver tissue at different time points (Supplementary Fig. S3a). The 41 modifications were m^5^U, m^5^C, C, A, G, U, 3’-OMeA, Cm, m^3^C, i^6^A, m^1^A, s^2^C, m^6^A, 3’-OmeC, Am, m^22^G, Um, I, m^1^G, m^7^G, m^2^G, 3’-OMeI, s^2^U, s^4^U, ac^4^C, 3’-OMeU, Ψ, Im, m^3^U, m^1^Ψ, t^6^A, ms^2^t^6^A, m^5^Cm, m^2,2,7^G, ac^4^Cm, 5’-OMeT, Gm, m^5^s^2^U, mo^5^U, hm^5^C, m^5^Um. All the modifications were accurately measured, as demonstrated in the ion channel map (Supplementary Fig. S3b). The variation in these modifications is shown in the heatmap and data (Supplementary Fig. S3c and Supplementary Table S2). In NS-KO cells, tRNA expression was reduced (Fig. 3e). Modifications with the most significant variations were tRNA m^5^U and the previously described m^5^C.^13^ Both are significantly decreased in the NS-KO sample (Fig. 3f, g).

The above findings showed that NS-KO functions by losing its methyltransferase activity while reducing tRNA m^5^U and m^5^C modifications. We then investigated whether NS-KO-derived small RNAs were sufficient to improve cell proliferation and survival under stress. We isolated liver RNA fragments of 14–50 nt and 50–100 nt (as a negative control) from WT and NS-KO mice (Fig. 3h). As the size of tsRNAs are always less than 50 nt, we considered 14–50 nt RNAs as tsRNAs-containing RNAs, and 50–100 nt RNAs as non-tsRNAs and negative control. We first detected the proliferative difference under low H_2_O_2_ stress between 14–50 nt RNAs and 50–100 nt RNAs. The EdU incorporation assay showed that the transfection with 14–50 nt RNA fragments from NS-KO mice increased cell proliferation instead of those fragments from WT mice (Fig. 3i, and Supplementary Fig. S3f). However, for 50–100 nt RNA fragments, we did not find any proliferative difference between WT mice and NS-KO mice (Supplementary Fig. S3d, f). Similarly, the CCK-8 assays showed that 14–50 nt RNA fragments from NS-KO mice increased cell survival after injury instead of 14–50 nt RNA fragments from WT mice and 50–100 nt RNA fragments from both mice (Fig. 3j, and Supplementary Fig. S3e, f). Thus, NS-KO-derived 14–50 nt RNA fragments play an essential role in improving cell proliferation and survival after injury.

### tRF-1s are the essential products of NS-KO

**Fig. 4:**
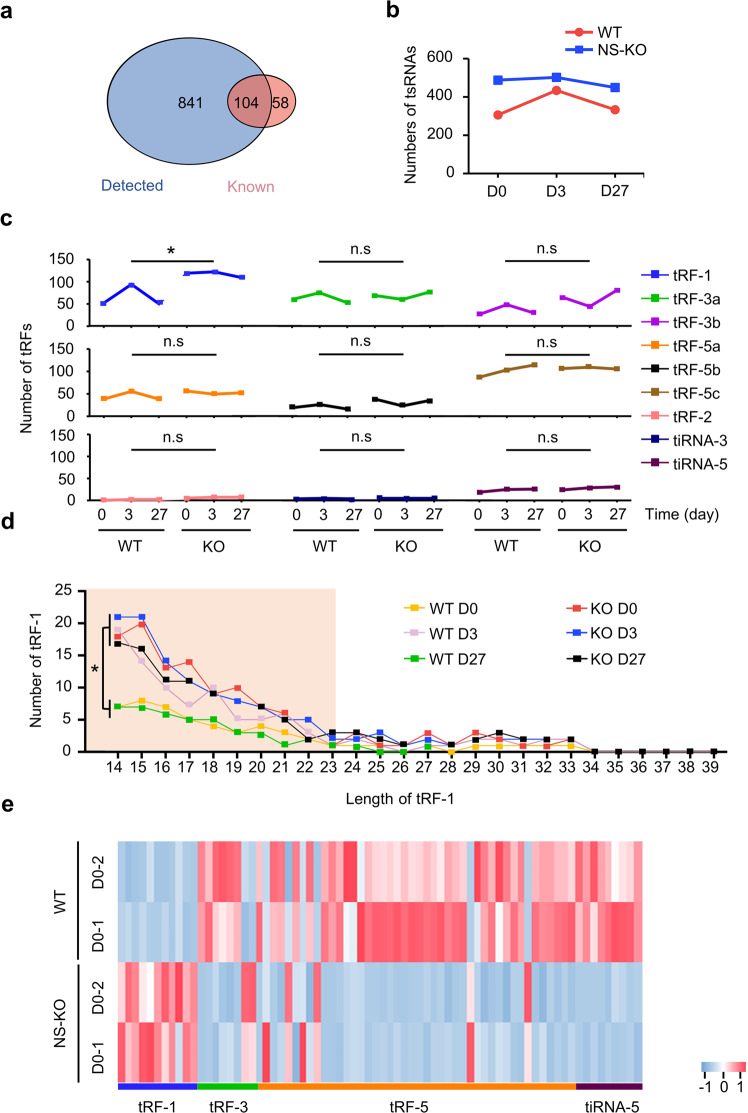
tsRNA-seq showing the signature of tRF-1s in WT and NS-KO mice subjected to stress. **a** Venn diagram showing the known tsRNAs from tRFdb^20^ and the tsRNAs detected from tsRNA-seq. **b** Group line chart showing the different number of tsRNAs at different time points in WT and NS-KO mice. D0: day 0: no injury; D3: day 3 after injury; D27: day 27 after injury. *n* = 2 mice. **c** Group line chart showing the distribution of subtypes of tsRNAs. The Y-axis represents the number of tsRNAs subtypes. The X-axis represents the injury time points in WT and NS-KO mice. **d** The number of tRF-1s against the length of tRF-1s reads. A line chart showing the sequence read length distribution in WT and NS-KO mice at different injury time points. The X-axis represents the length of tRF-1s, and the Y-axis represents the number of tRF-1s. The color represents the injury time points in WT and NS-KO mice. **e** Hierarchical clustering heatmap of differentially expressed tsRNAs (*P* < 0.05 and log_2_FC > 2). Each column represents each tsRNA, and all selected tsRNAs are categorized into no more than six clusters based on the tsRNAs subtypes. Each row represents a replicate. The color in the panel represents the relative expression level of the same type of tsRNAs (row normalization by z-score). The color scale is shown on the right: blue represents an expression level below the mean; while red represents an expression level above the mean. All data in this figure are acquired by tsRNA-seq, with two duplicates performed for each group. All data are expressed as the mean ± SD. *P < 0.05, *n.s* no significance, Student’s *t*-test

To achieve a comprehensive analysis of the metabolic characteristics of tsRNAs and analyze the tsRNAs differences in WT and NS-KO mice after different stress damage, we first determined that the number of miRNAs was not notably altered by NS KO (WT 52.96%, NS-KO 53.12%) (Supplementary Fig. S4a). Through tRNA-derived small RNA sequencing (tsRNA-seq), we identified 841 undocumented tsRNAs and 104 known tsRNAs documented in the tRFdb database (Fig. 4a). Then, we analyzed tsRNA expression by comparing samples derived from WT and NS-KO mice and identified 366 upregulated and 267 downregulated tsRNAs (Supplementary Fig. S4b), indicating that NS-KO caused more tsRNA upregulation than downregulation. Interestingly, tsRNAs in NS-KO mice remained at a relatively high level throughout the injury process, whereas tsRNAs peaked in the WT at D3 (Fig. 4b). Therefore, we assumed that day 3 was the most active time for hepatocyte proliferation and tsRNAs generation throughout the repeated injections of CCl_4_ (Figs. 2g, 4b), and NS-KO mice may have a “pro-repair tsRNA pattern” similar to WT D3 mice. These results demonstrated that *NSun2* loss increased tsRNAs in NS-KO mice and reserved sufficient tsRNAs to respond to stress damage in advance, but in WT mice, tsRNAs can only be produced at WT D3.

After further analyzing the tsRNAs landscape in WT and NS-KO mice at different stages of liver injury, we identified a sudden increase of tRF-1s in NS-KO mice compared with other tsRNAs. At all liver injury time points, there were significantly more tRF-1s in the NS-KO group than in the WT groups, but only increased on Day 3 in the WT groups (Fig. 4c, d). A detailed analysis of tRF-1s revealed that the length of tRF-1s was mainly between 14 and 23 nt (Fig. 4d). In general, the loss of *NSun2* mainly produced tRF-1s of 14–23 nt in the liver.

In-depth tRNA isodecoders analysis for tRF-1s, Arg-CCT, Cys-GCA, Gly-TCC, Ser-AGA, His-GTG, Val-CAC, Ile-AAT, Met-CAT, and Phe-GAA in the WT D3 and KO D0 groups revealed similar ascending trends (other low- or undetected isodecoders of tRF-1s are not listed) compared with the WT D0 group (Supplementary Fig. S4c). We also detected tRF-1s expression and found that it was higher in NS-KO mice than in WT mice, whereas the expression of other types of tsRNAs was lower in NS-KO mice than that in WT mice (Fig. 4e). On day 3, almost all the NS-KO mice had higher tRF-1s expression than WT mice (Supplementary Fig. S4d). The expression pattern of tsRNAs returned to the pre-injury pattern on day 27 (Supplementary Fig. S4e). Meanwhile, based on the differences between WT and KO and between WT D0 and WT D3, we identified 68 possibly functional tRF-1s (Supplementary Table S1). Therefore, we identified that tRF-1s were increased in NS-KO, analyzed the tRF-1s landscape, and identified 68 NS-KO-derived tRF-1s are candidate tsRNAs for preventing liver injury and responding to stress damage.

### Screened tRF-1 rescues liver injury in vitro and in vivo

**Fig. 5:**
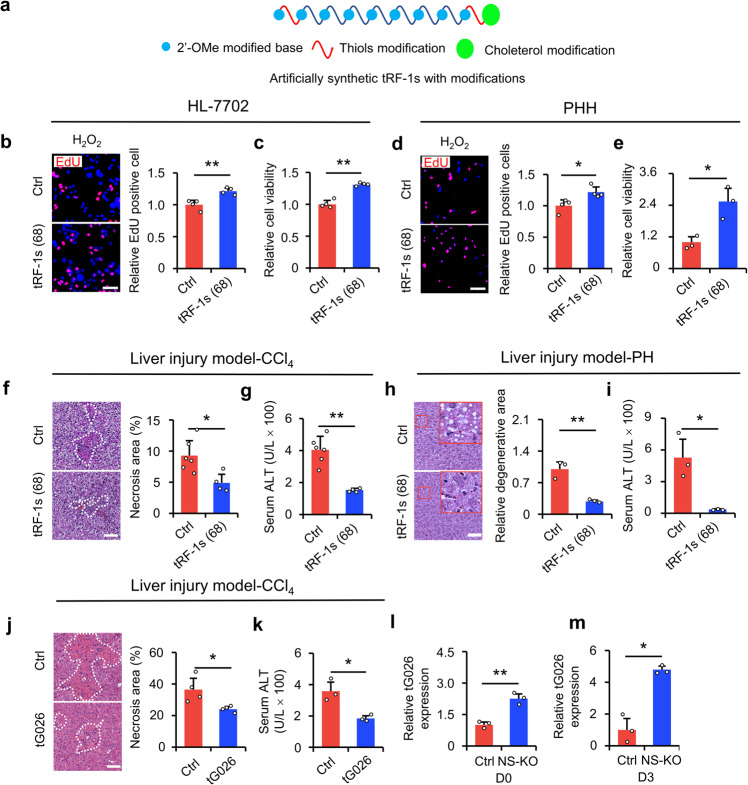
Screened tG026 promoted cell proliferation and survival after injury. **a** The modified pattern of artificially synthesized tRF-1s. **b** HL-7702 cells were transfected with 68 artificially synthesized tRF-1s. Random code sequence was used as a negative control. EdU-positive cells were analyzed relative to the control. *n* = 4 replicates. Representative images (left panel) and statistical results (right panel) are shown. Scale bar, 100 μm. **c** Relative cell viability (CCK-8 assay) was assessed by detecting the OD450 value. *n* = 4 replicates. **d** The results of (**b**) in PHH cells. *n* = 4 replicates. **e** The results of (**c**) in the PHH cells. *n* = 3 replicates. **f**, **g** The effects of injection of 68 synthetic tRF-1s with modifications in vivo in CCl_4_-induced liver injury. H&E staining (**f**) and the liver function index, ALT (**g**), were used to assess the extent of liver injury. Control mice, *n* = 6; tRF-1s mice, *n* = 4. **h**, **i** The effects of injection of 68 synthetic tRF-1s with modifications in vivo on partial hepatectomy liver. H&E staining (**h**) and the liver function index, ALT (**i**), were used to assess the extent of liver injury. *n* = 3 mice. **j**, **k** The effects of injection of synthetic tG026 with modifications in vivo on CCl_4_-induced liver injury. H&E staining (**j**) and the liver function index, ALT (**k**), were used to assess the extent of liver injury. H&E staining, *n* = 4 mice; ALT measurement, *n* = 3 mice. **l**, **m** The relative expression of tG026 in NS-KO mice at D0 (**l**) or D3 (**m**) after injury. D0: no injury; D3: on day 3 after liver injury. *n* = 3 mice. For data in this figure, control mice received an i.p. injection of random code sequence RNAs. Images shown are representative. Scale bar for H&E staining is 50 μm. All data are presented as mean ± SD. *P < 0.05, **P < 0.01, Student’s *t*-test

As described above, the by-products of liver injury are mainly tRF-1s. To elucidate whether tRF-1s could be a potential treatment for liver injury, we artificially synthesized 68 tRF-1s described in Fig. 4. We modified the tRF-1s with phosphorothioate (PS), 2’-O-methyl (2’-OMe), and cholesterol to improve their stability and transfection efficiency in vivo (Fig. 5a). Then, we transfected artificial synthetic tRF-1s into HL-7702 cells and primary human hepatocytes (PHH) and verified their functions in vitro and ex vivo. The transfection efficiency of tRF-1s was greater than 60% (Supplementary Fig. S5a). The EdU incorporation assay showed that the synthetic tRF-1s promoted cell proliferation (Fig. 5b). The CCK-8 assay showed that the cell viability improved after overexpression of tRF-1s (Fig. 5c). In PHH, we also acquired consistent results (Fig. 5d, e). To verify the function of the modified synthetic tRF-1s in vivo, we intravenously injected tRF-1s into mice with liver injury. First, we selected one tRF-1, tRF-Met-CAT-049, to determine the metabolic time-course of small RNAs in vivo. The results showed that the expression of tRF-Met-CAT-049 began on day 1 after injection and peaked on day 3. By day 4, the expression of injected small RNAs was maintained at relatively high levels (Supplementary Fig. S5b). Hence, we injected tsRNAs just once to cure the mice with liver injury because a high-level expression of injected RNAs could be maintained during this time.

The results of injecting 68 tRF-1s indicated reduced damage and improved liver function (Fig. 5f–i and Supplementary Fig. S5c, d). These results illustrated that 68 tRF-1s accelerated the recovery of injured liver cells in vitro and in vivo. Then, to further screen the most efficient tRF-1 from the 68 potential ones, we performed EdU staining in HL-7702 cells stressed with H_2_O_2_ for each potential tRF-1, and found that tG026 had the strongest ability to promote proliferation under stress (Supplementary Fig. S5e). The CCK-8 assay results showed that tG026 promoted cell survival under stress (Supplementary Fig. S5g). In another cell line, AML12, we demonstrated a similar result (Supplementary Fig. S5g, h). Overall, we showed that tG026, which was identified in our screening, ameliorated liver injury (Fig. 5j, k) and promoted cell proliferation after injury in vivo (Supplementary Fig. S5i), and tG026 expression was increased after NS-KO (Fig. 5l, m). In sum, through screening, we identified that tG026 could ameliorate liver injury as mentioned above.

### tRF-Gln-CTG-026 reduces global protein synthesis by regulating ribosome assembly

**Fig. 6:**
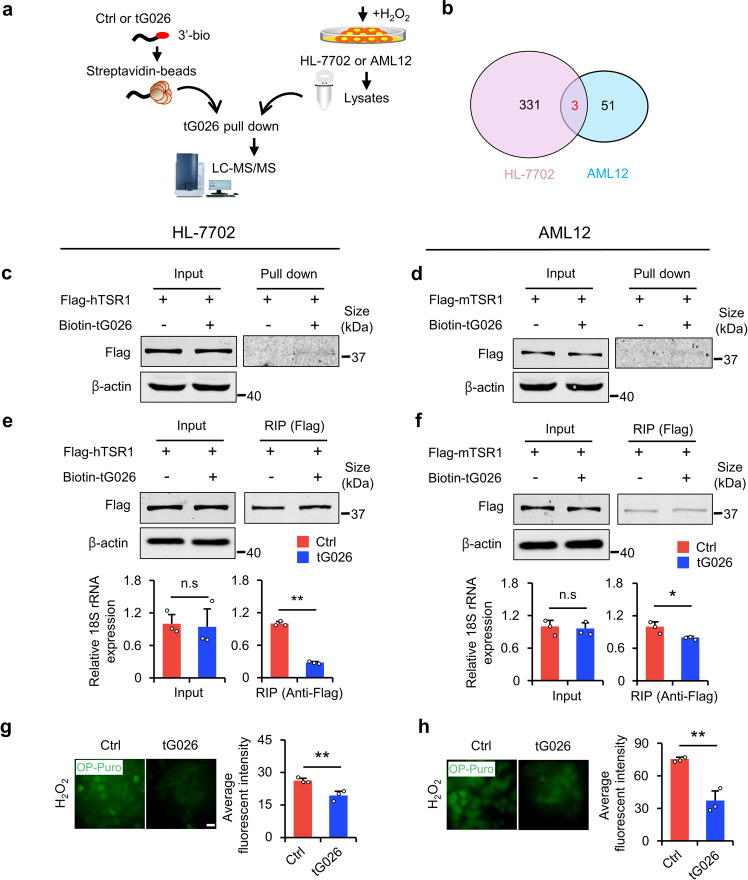
tG026 inhibits the interaction between TSR1 and 18S rRNA to reduce GPS. **a** The schematic of using RNA pull-down binding LC-MS to screen interactive proteins with tG026. **b** Venn diagram showing the interacting proteins with tG026 common to both HL-7702 and AML12 after tG026 RNA pull-down. The screening standards of interacting proteins are as follows: 1. The average value in control group must be zero; 2. The average value in tG026 group is non-zero. The red font represents the number of interacting proteins common to HL-7702 and AML12. *n* = 3 replicates. **c**, **d** RNA pull-down verified the interaction between TSR1 and tG026 in HL-7702 (**c**) and AML12 (**d**). **e**, **f** After tG026 and Flag-TSR1 were transfected, the interaction between TSR1 and 18S rRNA in HL-7702 (**e**) and AML12 (**f**) were detected by RIP. Input showing the unchanged 18S rRNA expression after transfection of tG026. *n* = 3 replicates. **g**, **h** Global protein synthesis under H_2_O_2_ stress in HL-7702 (**g**) and AML12 (**h**) was assayed by OP-Puro incorporation. Representative images (left panel) and statistical results (right panel) are shown. Scale bar, 50 μm. *n* = 3 replicates. All data are expressed as the mean ± SD. *P < 0.05, **P < 0.01, *n.s*. no significance, Student’s *t*-test

To explore the potential mechanism of tG026, we performed an RNA pull-down–LC-MS/MS assay to screen for proteins that interact with tG026 (Fig. 6a). After searching for the proteins that interacted with tG026 common to both the HL-7702 and AML12 cell lines, we chose pre-rRNA-processing protein TSR1 homolog (TSR1), which participates in Pre-40S ribosome assembly, for the subsequent experiments (Fig. 6b). We speculated that tG026 might regulate ribosomes. By the RNA pull-down assay, we verified the interaction between TSR1 and tG026 in both cell lines (Fig. 6c, d). To determine how tG026 regulates ribosomal assembly, we overexpressed tG026 in HL-7702 and AML12 cells (Supplementary Fig. S6a, b). The results suggest that tG026 did not alter 18S rRNA expression, but inhibits the association between TSR1 and 18S rRNA. This indicates that tG026 suppresses the interaction between TSR1 and the ribosome because 18S rRNA is an essential component of the ribosomal complex (Fig. 6e, f). Finally, tG026 inhibits GPS (Fig. 6g, h). Actually, as reported before, the loss of *NSun2* also reduces GPS in skin tissue,^15^ and we show that the loss of *NSun2* suppresses GPS in the liver (Supplementary Fig. S6c–f). Hence, the loss of *NSun2* reduces GPS through generating tG026. We then used another inhibitor of protein synthesis, cycloheximide (CHX) (Supplementary Fig. S6g, h), to determine the roles of GPS in injury. The results show that CHX promotes survival under high damage stress (Supplementary Fig. S6i, j). However, it actually reduces cell proliferation under low damage stress owing to toxicity (Supplementary Fig. S6k, l). Collectively, the loss of *NSun2* causes the generation of tG026. tG026 relieves GPS by repressing the association between TSR1 and 18S rRNA to promote proliferation and survival after liver injury and can be developed as an RNA medicine to alleviate liver injury.

## Discussion

**Fig. 7:**
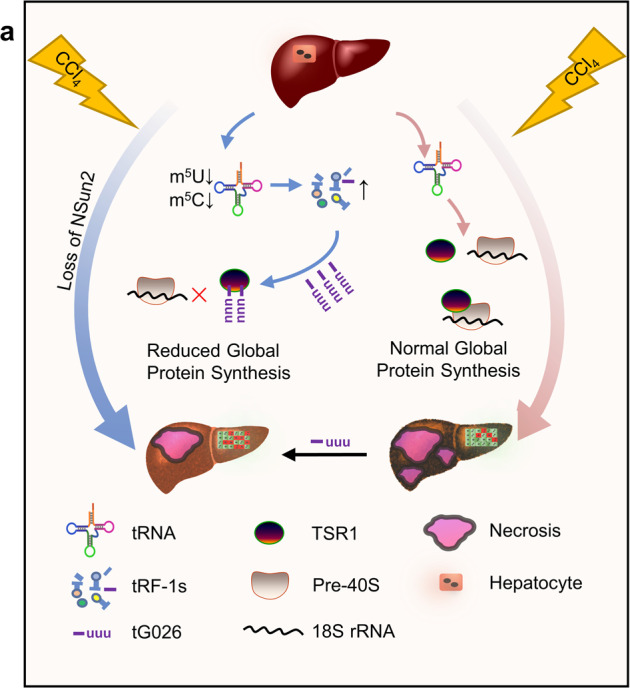
Model of tG026 mechanism in liver injury. Loss of NSun2 can ameliorate liver injury produced by CCl_4_ injection. Mechanistically, the loss of NSun2 reduces m^5^U and m^5^C modifications. Hence, the tRNAs become unstable and produce tsRNAs, particularly tRF-1s. Through further screening experiments, tG026 was found to inhibit the association between TSR1 and 18S rRNA by interacting with TSR1, thereby reducing global protein synthesis and alleviating liver injury

Our study has elucidated that a new tRF-1 (tG026) screened from NS-KO-derived tsRNAs ameliorates liver injury through the suppression GPS, and our hypothesis is summarized in Fig. 7. It is known that protein synthesis is a very energy-consuming process. A high level of global protein synthesis requires a large quantity of energy and causes endoplasmic reticulum (ER) stress, which can initiate “lethal synthesis” under stress conditions.^18^ Furthermore, ER stress can also lead to the accumulation of misfolded and unfolded proteins, which triggers apoptosis or downstream stress signaling, e.g., the death signal program induced by PERK-mediated eIF-2α phosphorylation.^21^ The inhibition of GPS can reduce energy consumption, relieve ER stress, and increase the purge of unfolded and misfolded proteins.^22^ Hence, the inhibition of GPS is a protective reaction against stress to save energy and improve proliferation and survival. Because of toxicity, effectiveness, or adverse effects, the strategy to inhibit GPS has not been used to improve liver injury.^23^

Previous studies have suggested some adverse effects during the process of tissue injury and repair. Long-term stimulation of tsRNAs induces the activation of tumor-related signals, promotes proliferation and survival of tumor cells, and finally accelerates tumor progression.^8^ However, these studies mainly focus on tsRNAs other than tRF-1s. There are currently few studies on tRF-1s, and very limited research investigates the regulative role of tRF-1s in cell proliferation and the Ago complex.^5,24,25^ There are an abundance of different types of tsRNAs in the liver. As distinctive small RNAs, different tsRNAs regulate gene expression through different mechanisms. tsRNAs can bind to the coding sequence (CDS), untranslated region (UTR), or ribosomes to regulate the expression of relevant genes. tsRNAs can also bind to Argonautes or PiwiL2 proteins to function as miRNAs or piRNAs^26^ and bind ribosomal mRNAs to enhance gene expression.^10^ A comprehensive and in-depth study of these therapeutic RNAs may enhance the treatment of liver diseases.

Traditional treatments for severe or end-term liver disease, including surgery, transplantation, some conventional pharmacological modalities, are characterized by poor prognoses, severe damage, and toxic and off-target effects. RNA therapeutics is generally the use of small RNAs as candidates to alleviate illnesses. This type of therapy, which is an improvement upon traditional therapies, has been introduced for liver disease. The most important aspect of RNA therapeutics is the introduction of new functional and safe RNA molecules with high efficiency. Interestingly, recent progress has introduced another class of small RNAs—tsRNAs, which are essential regulators in the stress response.^5,6,27^ Nowadays, larger numbers of tsRNAs are screened, and their corresponding mechanism and function have gradually been clarified. However, among all types of tsRNAs, few studies focused on tRF-1s have been reported. tRF-1s, cleaved from pre-tRNA, are similar to miRNAs in size and can be easily synthesized in vitro. Thus, theoretically, tRF-1s might be new targets that can be employed as clinical RNA therapeutics for liver disease. However, no tRF-1s or even other tsRNAs, have been developed to treat disease in clinical settings. In this study, we referred to ASO (antisense oligonucleotides) and siRNA design principles for the in vivo delivery of tG026^28^ which bring about improved liver accumulation. Our results suggest that the new tRF-1 (tG026 found from NS-KO) is a potential therapeutic target, accelerating the recovery of injured liver both in vitro and in vivo, and can be used as a potential marker for the diagnosis and prognosis of liver injury.

As the body’s largest metabolic organ, the liver has an abundance of tRNAs and ribonucleases. When the liver suffers an injury, the tRNA metabolism rate becomes faster, making tRNA more susceptible to cleavage by ribonucleases, resulting in the generation of many tsRNAs. Our results showed that the production of tsRNAs, especially tRF-1s, was abundant after liver injury (Fig. 4c, e). ELAC2, responsible for cleaving the 3’ end of pre-tRNAs to form tRF-1s, is homologous to the endonuclease RNase Z from *Saccharomyces cerevisiae*. The expression of ELAC2 was increased by 22.4-fold during the rat liver repair process, and ELAC2 was one of the most highly expressed genes identified from the microarray analysis, which may be the reason for increased tRF-1s after liver injury. The function, types, and expression of NS-KO-derived tsRNAs differ across different tissues or cells, suggesting a tissue-dependent regulation for NS-KO-derived tsRNAs. Several studies show that NS-KO causes hypomethylated tRNAs, increases 5’ tRNA fragments, and reduces global protein synthesis. Functionally, NS-KO inhibits cell differentiation and increases cell sensitivity to cytotoxic drugs.^13,29^ In contrast, in *S. cerevisiae*, the loss of *Trm4a*, the homolog of *NSun2*, promotes cell resistance to CaCl_2_ stress.^30^ For fruit flies, although NS-KO causes a decrease in tRNA abundance, the tsRNAs abundance does not increase, indicating that NS-KO does not necessarily increase the accumulation of 5’ fragments. NS-KO might only reduce the tRNA abundance instead of altering tsRNAs abundance.^31^ Our study found an increase in tRF-1s and an improvement in cell survival and proliferation in the NS-KO liver injury model. Therefore, in NS-KO, different tissues or cells might generate different tsRNAs, which would have other functions.

tRNA modifications are generally stable but change with various damage stresses, resulting in changes in tRNA stability, tsRNA generation, ribosome binding capacity, misreading, and frameshift. The regulation of tRNA modifications is also involved in many biological processes, such as cell proliferation, survival, and metabolism.^32^ In our study, in NS-KO, in addition to m^5^C, which has been reported previously, m^5^U in tRNA was also significantly changed. After injury, many other tRNA modifications occurred to different degrees (Supplementary Fig. S3c and Supplementary Table S2), which indicated that these tRNA modifications play essential roles in liver injury and repair. The deletion of various modification sites will cause different cleavage positions, resulting in different types of tsRNAs. Accumulated evidence shows that NSun2 could catalyze m^5^C at C34 in pre-tRNAs, and the lack of this site facilitates the enzymatic cleavage of tRNAs by angiogenin.^13^ However, the cleavage of angiogenin and loss of C34 do not generate tRF-1s. Hence, the production of tRF-1s might be related to the loss of newly discovered m^5^U modifications or non-C34 m^5^C modifications in tRNA (Fig. 3h, i). As NSun2 can modify the precursor tRNA, m^5^U modification is also likely to alter the precursor tRNA; therefore, the co-deletion of these two modifications may cause significant change in tRF-1s.

The relationship between global protein synthesis and liver injury is complex. We could not find an effective method to perform a rescue experiment for global protein synthesis. Instead, we used another protein-reducing reagent (CHX) to identify the reparative effect of reducing global protein synthesis in the process of severe injury. However, owing to the toxicity of this reagent, the use of CHX suppresses cell proliferation under stress (Supplementary Fig. S6k, l). Because of the technical restriction, there are no established editing methods for tRNA modification at a specific location. Therefore, we could not show further associations between tRF-1s and the m^5^U or m^5^C modification. In-depth examination and editing of these modifications and tsRNAs will help to extend our understanding of the molecular mechanism of liver injury and lead to the development of new treatment methods for liver injury.

## Conclusion

In our study, the utilization of tRNA-degraded product RNA that regulates GPS reduced RNA toxicity. The associated mechanism was through the tG026-mediated disruption of the interaction between TSR1 and 18S rRNA, which occurred because of the interaction of tG026 with TSR1, stopping the association between TSR1 and Pre-40S ribosome. This causes inhibition of the ribosome assembly program, which reduces GPS.

## Materials and methods

### Experimental model and subject details

For this study, mice from a B6D2F1/Crl background were purchased from Charles River. The mice were kept in a specific-pathogen-free environment in the Animal House of the Institute of Zoology, Chinese Academy of Sciences. The environment consisted of artificial light for 12 h in the daytime and a 12-h night (lights off, 7 p.m. to 7 a.m.). The mice could eat and drink freely. Male littermates of a similar age and weight were used in the liver injury experiments to allow the comparison of the results in the experiments. All animal experiments in this study followed local rules, procedural rules, and ethical criteria set by the local experimental animal ethics committee and the Institutional Animal Care and Use Committee (IACUC) at the Institute of Zoology, Chinese Academy of Sciences.

### Liver injury mice

Single or repeated injections of CCl_4_ (Sigma) were performed to induce acute and long-term liver injuries, respectively.^33^ Corn oil was used to dilute CCl_4_ to form a 40% CCl_4_ solution, which was then intraperitoneally injected into 9–12-week-old male mice at 2 mL kg^−1^ body mass. The mice were sacrificed at the indicated time points, and serum was isolated for liver function tests. Entire liver tissues were separated to analyze liver injury, including necrosis via hematoxylin and eosin (H&E), fibrosis via Sirius Red staining, gene expression analysis via quantitative real-time polymerase chain reaction (qRT-PCR) or western blotting, proliferation by Ki67 and BrdU, apoptosis by TUNEL staining, and next-generation sequencing was performed by KangCheng Biotech Corporation.

### Oligonucleotide transfection

For *NSun2*-specific inhibition, *NSun2* siRNA1 (stealth HSS123462), siRNA2 (stealth HSS182713), and control siRNA (stealth 12935-300) were purchased from Invitrogen (see Supplementary Table S3 for sequences). A concentration of 25 pmol was used for each transfection in accordance with the manufacturer’s instruction and Lipofectamine RNAiMAX was used for transfection.

### Culture cells and transfection

The human cell line HL-7702 (CL-0111) and mouse liver cell line AML12 (AML12) were purchased from Procell. HL-7702 cells were cultured in RPMI 1640 medium (GIBCO, 72400120), and AML12 cells were cultured in AML12 Special Medium (CM-0602). Human primary liver cells were purchased from BioreclamationIVT (M00995) and cultured in HM medium, as reported elsewhere.^34^ The medium was changed every other day or every day based on cell density. HL-7702 and AML12 were digested with 0.25% trypsin including EDTA (GIBCO, 25200056) every other day or every 3 days, and passaged. Cells were identified by STR. For cell transfection, a mixture of *NSun2* siRNAs (HSS123462 and HSS182713, 1:1) and control siRNA were transfected with Lipofectamine RNAiMAX Reagent (Thermo, 13778-150) in accordance with the manufacturer’s instructions. When siRNAs and plasmids were co-transfected, Lipofectamine 3000 reagent (Thermo, L3000015) was used as specified by the manufacturer’s guidelines. For the transfection of NS-KO mice-derived small RNAs, 50–100 nt RNAs were used as controls, and 14–50 nt RNAs were sampled. When transfected, these RNAs were mixed with BLOCK-iT Fluorescent Oligo (Thermo, 2013) in a 3:1 ratio, which was used as a marker of transfection efficiency. Lipofectamine 3000 reagent was used as the transfection reagent. For the transfection of synthesis tsRNAs, these tsRNAs were mixed in equal proportions, and the mixtures were mixed with BLOCK-iT Fluorescent Oligo in a 3:1 ratio. For six-well plates, 80 pmol RNAs was used for transfection. Lipofectamine 3000 was used as the transfection reagent. For the tsRNA transfection method, refer to the siRNA transfection method.

### Proliferation assay

Cultured HL-7702 cells were exposed to 10 μM H_2_O_2_ (AML12 10 μmol, PHH 30 μmol) for 48 h after the cells were transfected with siRNAs or plasmids for 24 h (or 10 μg/mL CHX for 1 h). Then HL-7702, AML12, or PHH cells were incubated with 50 μM 5-ethynyl deoxyuridine (EdU) for 3 h, and washed once with PBS. The cells were fixed with 4% PFA in PBS for 0.5 h. Paraformaldehyde was neutralized for 5 min with 2 mg/mL glycine. The cells were washed for 5 min with PBS, and 0.5% Triton X-100 (in PBS) was added to the cells for 10–15 min. The cells were washed for 5 min with PBS. Subsequently, the cells were stained with Cell-Light Apollo 567 Stain Kit (RiboBio, C10310-1) in accordance with the manufacturer’s instruction in dim lighting at room temperature and the nuclei were stained with Hoechst 33342. Representative images were acquired using an inverted fluorescence microscope (LEICA, DM2500).

### Cell viability

Cell viability was determined with the Cell Counting Kit-8 (CCK-8, Dojindo, CK17) via a method in line with the manufacturer’s manual. In this assay, cell viability was reflected indirectly by the detection of dehydrogenase activity, which is positively correlated with living cell count. HL-7702, AML12, or PHH cells were transfected with siRNAs or plasmids. After transfection (48 h for siRNAs or 24 h for plasmids, or treatment with 10 μg/mL CHX for 1 h), HL-7702 cells were treated with RPMI 1640 containing 500 μM H_2_O_2_ for 1 h or 8 mM CCl_4_ for 10 h, 20 mM acetaminophen for 12 h, or mixed lipidic acids (0.25 mM oleic acid + 10 μg/mL palmitic acid) for 24 h in 5% CO_2_ and 37 °C. AML12 cells were treated with AML12 medium containing 100 μM H_2_O_2_ for 2 h. PHH cells were treated with HM medium containing 500 μM H_2_O_2_ for 2.5 h. After the induction of cell damage, medium containing 10% CCK-8 was added to the cells. The culture dish was kept in the incubator under 5% CO_2_ and 37 °C. The absorbance value at 450 nm was measured in a 96-well culture plate at the indicated time points (Synergy 4, Bio-Tek).

### Western blotting

To obtain proteins from the mouse liver, the livers were ground in liquid nitrogen, and RIPA lysis buffer (Sigma) supplemented with protease inhibitor (Roche) was added to the livers to lyse the tissue, which were then homogenized and centrifuged at 4 °C and 12,000 × *g* 10 min. For cultured cells in vitro, RIPA buffer containing protease inhibitor was added to the culture dish to extract cell proteins in the culture dish. The cells layers were scratched and transferred to Eppendorf tubes. The cells were lysed in ice for 30 min, and then centrifuged at 4 °C and 12,000 × *g*. Protein concentration was determined using the Pierce BCA Protein Assay Kit (Thermo Fisher). The proteins were separated by SDS-PAGE. Equal amounts of protein were added to each well of the gel. The proteins were transferred to nitrocellulose filter membranes. Western blotting was performed as reported previously.^35^ The following primary antibodies were used: mNSun2 (1:1000, Abcam, 128243); β-actin (1:2000, Abcam, AB8226); hNSun2 (1:500, Proteintech, 20854-1-AP); puromycin (1:100, Millipore, MABE343); flag (1:2000, Cell Signaling Technologies, 14793S). Fluorescence-conjugated secondary antibodies were purchased from Abcam (1:10,000, Abcam).

### Wound-healing assays

*NSun2* siRNA or control siRNA was transfected into HL-7702 cells. After 24 h, the cells were exposed to H_2_O_2_ (10 μM), cultured for a further 24 h, and then subjected to a wound-healing assay. The wound-healing assay was performed in accordance with Ibid’s instructions for the Culture-Insert 2 Well (Ibid, 80209). Briefly, cells were treated with mitomycin C for 2 h to inhibit cell proliferation. Dead cells and clean cell debris were removed. The cells were digested and counted. The cell suspension was then prepared, and 70 μL was applied to each well (when the cells were approximately 100% confluent) while avoiding shaking. The cells were incubated at 5% CO_2_ and 37 °C to allow the cells to adhere to the culture plate. After the cells were attached and stretched, the Culture-Insert 2 Well was carefully and gently removed from the well with a sterile tool. The experimental well was filled with the indicated medium, and the cells were allowed to migrate for 0 or 15 h. Representative images were acquired at the indicated time using an inverted microscope (LEICA, DM2500).

### *NSun2* knockout mice

To knock out the *NSun2* gene, gRNA oligos targeting this gene were constructed into a vector plasmid with the U6 promoter. Specific gRNA sequences were designed using the website of Zhang Lab (http://www.genome-engineering.org/crispr/). Six sgRNAs (sgRNA1–sgRNA6) were created to target six mouse *NSun2* (*mNSun2*) gene positions and eventually two sgRNAs were screened. Then, 3 μg of Cas9-GFP plasmid and 2 μg of sgRNA plasmid were electroporated into mouse embryonic fibroblast cells (MEFs) in a 3.5 cm dish. GFP-positive cells were obtained three days later by fluorescence-activated cell sorting (FACS). The genomic DNA of these GFP-expressing cells was extracted and amplified using the Mouse Direct PCR Kit (Biotool, B40015). PCR products were analyzed by the T7 endonuclease I (T7EI) assay (NEB, M0302). Simultaneously, PCR products were cloned into PMD18-T and transformed into DH5α competent cells. Subsequently, 20 colonies were selected for each sgRNA and sequenced using the Sanger method, and the knockout efficiency for each sgRNA was calculated. Primers for the *NSun2* locus are shown in Supplementary Table S3. Finally, sgRNA1 and sgRNA2, for which the efficiencies were greater than 60%, were selected (see Supplementary Table S3 for sequences). sgRNA1, sgRNA2 (NEB, E2040S), and Cas9 mRNA (NEB, E2060S) were transcribed in vitro and purified (Omega, R6247-010).

Cas9 mRNA/sg*NSun2* was delivered by pronuclear microinjection. The concentration was 100/50 ng/μL. The knockout of *NSun2* in mouse embryos was produced through zygotic injection, and relatively high-efficiency transfection of mouse embryos of *NSun2* bi-allelic mutations were obtained. Eventually, the Δ56 bp mice were acquired for subsequent research. Our group directly deposited all relevant plasmids. Finally, *NSun2*-deficient mice were bred into a B6D2F1/Crl background and maintained by heterozygous mating.

### Hematoxylin and eosin staining

Fresh tissue was fixed with fixative (4% PFA) for more than 24 h, dehydrated with a graded ethanols, and dipped in wax. The wax blocks were cut into 4 μm thick slices. Paraffin sections were dewaxed and soaked in water. The sections were then immersed in Harris hematoxylin (Servicebio, G1005) for 3–8 min. The sections were washed and differentiated by 1% hydrochloric alcohol for several seconds. After rewashing the sections, 0.6% ammonia water was used to return the nuclei to blue. Slices were stained in eosin staining buffer for 1–3 min. The sections were then dehydrated until transparent and then sealed with neutral gum. Images were collected under a microscope, examined, and then analyzed.

### Examination of liver function

Mice blood was collected, stored at 4 °C overnight, and centrifuged for 15 min at 3000 × *g*. The supernatant was aspirated and the corresponding biochemical indices were measured. The liver enzymes ALT, AST, and ALP were measured. Measurements were performed using an automatic biochemistry analyzer (Chemray 240) with standard procedures.

### Immunofluorescence and immunochemistry staining of paraffin-embedded liver sections

Paraffin sections were made as described for the H&E staining method. The tissues were fixed in 4% PFA. The paraffin sections were dewaxed and soaked in water. The tissue slices were placed in a box containing EDTA buffer (pH 8.0) to perform antigen repair in a microwave oven, circled with a histochemical pen, and autofluorescence quenching was performed. BSA was dropped in the circle and incubated for 30 min. A primary antibody was added, and the slices were incubated in a wet-box at 4 °C overnight. A secondary antibody was added, and the sections were incubated for 1 h in dim light at room temperature. The nuclei were counterstained with 4,6-diamidino-2-phenylindole (DAPI) or hematoxylin. After the slices were slightly dried, anti-fluorescence quenching sealing buffer was applied to the sections.

The proliferation of hepatocytes in vivo was detected by the BrdU incorporation assay. Mice were administered an intraperitoneal injection of BrdU (B5002, Sigma) solution (50 μg BrdU/g mouse body weight) 3 h before they were euthanized. Next, the paraffin sections were dewaxed, soaked in water, and repaired with citric acid buffer (pH 6). HCl (1 N; 1 mol/L) was added and the solution was heated at 37 °C for 30 min to denature DNA. The sections were fixed with 4% paraformaldehyde solution for 10 min to prevent DNA renaturation. The section was circled with a histochemical pen, and autofluorescence quenching was performed. BSA was dropped in the circle and incubated for 30 min. Primary antibody and secondary antibody were added. The nuclei were counterstained with DAPI, and the section was sealed.

Hepatocytes apoptosis in vivo was measured using the TUNEL assay in accordance with the instructions of the In Situ Cell Death Detection Kit (Roche, 11684795910). Paraffin sections were dewaxed to water. Repair: Protein K working solution was dropped in the circle to cover the tissue and the sections were held at 37 °C for 25 min and washed three times with PBS. To destroy the membrane, the appropriate solution was added, and the tissues were covered and kept for 20 min at room temperature. TdT and dUTP were mixed in a 1:9 ratio. The mixture was incubated in a circle to cover the tissue at 37 °C for 2 h. The nuclei were counterstained with DAPI, and the sections were sealed.

The primary antibodies were diluted as described below: rabbit polyclonal anti-NSun2 (1:200, Abcam, 128243), rabbit polyclonal anti-Ki-67 (1 μg/mL, Thermo, PA5-19462), and sheep polyclonal antibody to BrdU (10 μg/mL, Abcam, ab1893). Alexa Fluor 488- and Alexa Fluor 594-conjugated secondary antibodies (Thermo) were used in a dilution ratio of 1:1000. Neutral gum was used to seal the sections. Images were extracted using a microscope, collected, and analyzed. Fluorescent images were captured using an LSM 780 confocal microscope.

### Quantitative RT-PCR (qRT-PCR)

Tissue or cells were lysed using 1 mL TRIzol reagent (Invitrogen). Then, 200 μL of chloroform was added and shaken to homogenize the lysates, which were then centrifuged at 12,000 × *g* for 10 min at 4 °C. The upper aqueous phase was collected, and an equal volume of 70% ethanol was added. Total RNA was extracted using the PureLink RNA Mini Kit (Invitrogen, 12183020). RNase-free DNase (QIAGEN, 79254) was added to reduce DNA contamination. cDNA was obtained by a reverse transcription reaction using the Superscript Reverse Transcriptase Kit (Invitrogen). The detection of cDNAs for the indicated genes was completed by qRT-PCR. qRT-PCR was conducted in the AGILENT MX3005P equipment using the SYBR method utilizing Power SYBR Green PCR Master Mix (ABI, 4367659). Samples were normalized to β-actin or U6 expression via 2^−ΔΔCT^ calculation. The detection of tRF-Met-CAT-049 and tRF-Gln-CTG-026 was performed by a specific reverse transcription primer. After RNAs were extracted, RNAs were reverse transcribed with a specific primer. The primers for the detected genes are listed in Supplementary Table S3. All kits were utilized in line with the manufacturers’ instructions.

### Sirius red staining

First, the paraffin sections were dewaxed to water and then placed into saturated picric acid–Sirius Red staining solution (Servicebio, G1018) for 8 min. The sections were rinsed with anhydrous alcohol for several minutes and observed under a microscope until satisfactory. Then, the sections were baked in a 60 °C oven and subsequently made transparent in xylene for 5 min. The sections were sealed using neutral gum. Images were obtained using a microscope, collected, and analyzed.

### Plasmid vector construction

To investigate the function of NSun2, the specific human *NSun2* CDS (NM_017755.6) was amplified from the cDNA library of 293T cells using Tks Gflex DNA Polymerase (Takara, R060A). The sequence was sub-cloned into the pCDH plasmid vector (SBI, CD521A-1) containing the EF1α promoter, followed by producing the nonsense mutated pCDH-h*NSun2* so that it could not be targeted by stealth RNA. Meanwhile, *NSun2* point-mutated sequences C271A and K190M were amplified from the PCDH-h*NSun2* plasmid using *NSun2* C271A and K190M point-mutant primers. Similarly, these two point mutants were also sub-cloned into the PCDH vector. Huigene Biotechnology Corporation-synthesized human or mouse TSR1 cDNA and then was sub-cloned to pCDNA 3.0-Flag vector.

### Detection of tRNA modification based on LC-MS

First, we measured the concentration and integrity of the RNA samples. Subsequently, over 5 μg of total RNA for each sample was separated by electrophoresis with 7.5% PAGE containing 7 M urea. The 60–90 nt tRNA band was excised and RNAs were extracted with 0.3 M NH_4_Ac, glycogen, and ethanol. Purified tRNA was quantified using a NanoDrop ND-1000. Next, purified tRNA from the previous step was hydrolyzed to a single nucleoside and then dephosphorylated in a 50 μL reaction volume containing 10 U Benzonase (Sigma), 0.1 U phosphodiesterase I (US Biological), and 1 U Alkaline Phosphatase (NEB). The reaction was incubated at 37 °C for 3 h. Next, the pretreated nucleoside solution was deproteinized. Spin filters (10 kDa MWCO; Sartorius) were cleaned, and the hydrolyzed RNAs were then added to the cleaned spin filters (16,000 × *g* for 10 min, 4 °C). The filtration was collected for downstream LC-MS analysis. A mass spectrometer (Agilent 6460 QQQ) conjugated with an HPLC instrument (Agilent 1260), SB-Aq 3.5 μm 2.1 × 150 mm HPLC column (Agilent), was used to analyze mixed nucleosides. In addition, multiple reaction monitoring mode (MRM) were chosen. Briefly, the single nucleoside mixture was injected from tRNA into the LC-MS instrument. The HPLC conditions were set up following the reagent gradient below (Solution A, add a proper amount of formic acid into HPLC grade water to prepare formic acid solution; Solution B, add a proper amount of formic acid into 100% acetonitrile to prepare the formic acid solution). T. Time (min): 0.0, 5.0, 15.0, 15.1, 20.0, 20.1, 30.0. Solution A (V/V): 99.9%, 99.9%, 90%, 40%, 0%, 99.9%, 99.9%. Solution B (V/V): 0.1%, 0.1%, 10%, 60%, 100%, 0.1%, 0.1%. Flow (mL/min): 0.35, 0.35, 0.35, 0.35, 0.35, 0.35, 0.35. The mass parameters were as follows: nebulizer gas pressure, 40 psi; gas flow, 7 L/min; capillary, 4000 V (positive); gas temperature, 325 °C; sheath gas flow, 10 L/min.

Agilent Qualitative Analysis software was used to analyze LC-MS data. Briefly, MRM peak information of modified nucleosides for each sample was extracted. If the signal-to-noise ratio of the peak is <10, it was considered to be a measurable nucleoside. Peak areas were then corrected or normalized by the purified tRNA quantity of samples. This experiment was performed by Aksomics Corporation. The protocol used is from this corporation.

### Small RNA extraction and isolation

Isolate *NSun2* KO mice liver and gall bladder were removed, and the liver was cut into small pieces and placed in RNAlater (Thermo). Liver tissues were ground in liquid nitrogen after the lysis buffer was added. RNA no longer than 200 nt was extracted with a mirVana miRNA Isolation Kit (Thermo, AM1561) used in accordance with the manufacturer’s instructions. Next, 15% Novex TBE-Urea Gels (Thermo, EC68852BOX) (acrylamide:bis-acrylamide = 19:1, including 7 M urea, dissolved in 1 × TBE). Using 1 × TBE as a running buffer, TBE-U gels were run for 10–15 min to heat them to approximately 50 °C. Mix <200 nt RNAs and Gel Loading Buffer II (Thermo, 8547) at a 1:1 ratio (V/V) and heated at 95 °C for 5 min. The mixture was added to the loading well of the gel and run in the Mini Gel Tank (Thermo, A25977) until the blue band was 2 cm away from the bottom of the gel. Using the markers, the bands from 14 to 50 nt and from 50 to 100 nt were excised. To extract the small RNAs from the excised gel, 10 volumes of 1 M NaCl were added to the gel. The gel was ground with a syringe piston, and the crushed gels were rocked continuously at 4 °C overnight, centrifuged at 2000 × *g* for 5 min, and the supernatant was added to a new tube. The 14–50 nt and 50–100 nt RNAs were, separately purified using the mirVana miRNA Isolation Kit in line with the manufacturer’s manual. After the concentration of small RNAs was detected, they were stored at −80 °C.

### Construction of tsRNA sequencing library

Small RNA libraries were constructed from the livers of CCl_4_-induced mice stored in RNAlater. This sequencing used two independent biological replicates for each sample. First, we checked the integrality and concentration of total RNA samples and pretreated total RNA samples to remove some modifications hindering the construction of the tsRNA library. For example, demethylation of m^3^C and m^1^A could enhance the reverse transcription; 5’-adapter ligation is needed to phosphorylate 5’-OH (hydroxyl group) to become 5’-P; 3’-adapter ligation is needed to remove the 3’-cP (2’,3’-cyclic phosphate); and deacylated charged 3’-aminoacyl converts it to 3’-OH. After all RNA samples were pretreated, library preparation of tsRNA sequencing was conducted using the NEBNext Multiplex Small RNA Library Prep Set for Illumina (E7300L). Library preparation procedures included: (a) ligate 5’-adapter, (b) ligate 3’-adapter, (c) synthesize cDNAs, (d) amplify sequence by PCR, and (e) select 134–160 bp amplified products by PCR, which were the original 14–40 nt tsRNAs before amplifying. The Agilent 2100 Bioanalyzer was used to accurately assess the concentration and quality of the sequenced library to quantify the completed libraries. Referring to the quantitative results, the libraries were mixed in equal amounts. The mixture was sequenced using the corresponding instrument. After the libraries were mixed well, 0.1 M NaOH was used to denature the DNA fragments in libraries so that single-stranded DNA (ssDNA) molecules could be formed. The 1.8 pM ssDNA molecules mentioned above were loaded onto the reagent cartridge. The NextSeq system (Illumina NextSeq 500) was used to analyze the sequenced samples using the NextSeq 500/550 V2 kit (Illumina, #FC-404-2005) following the manufacturer’s guidelines. For sequencing, 50 cycles were performed. Aksomics Corporation performed this experiment in line with their protocols.

### Analysis of tsRNA-seq data

The Illumina sequencer first generated the raw data files (FASTQ format), and FastQC examined the sequencing quality.^36^ Using quality filtering and real-time base calling (Solexa pipeline version 1.8, Off-Line Base Caller software, version 1.8), clean reads were acquired from the raw sequence data generated from the Illumina NextSeq. The clean reads were recorded in FASTQ format. Subsequently, after Illumina quality control was performed, the sequencing reads were 5’, 3’-adapter trimmed directly from the clean reads, and the discarded reads (length <14 nt or length >40 nt) with cutadapt were recorded as trimmed reads^37^ in FASTA format. Trimmed reads passing Illumina Quality Control were aligned to allow for one mismatch only to the mature tRNA sequences, and then reads that do not map were aligned to allow for one mismatch only to precursor tRNA sequences using bowtie software.^38^

The expression profiling of tsRNAs and miRNAs was calculated based on counts of mapped reads; these counts were normalized as counts per million of total aligned reads (CPM). If the CPM was less than 20 in all samples, tsRNAs were filtered. The differentially expressed tsRNAs were screened based on the count value with the R package edgeR.^36^ The R language was used to compute statistics for the generation of Venn plots, scatter plots, and heatmaps according to expressed tsRNAs. Among them, the Venn diagram is based on the number of commonly expressed and differentially expressed tsRNAs. This diagram shows the number of tsRNAs expressed in all experimental groups and indicates the number of differentially expressed tsRNAs. The commonly expressed tsRNAs represent the CPM values that were greater than 20 in all experimental groups. The differentially expressed tsRNAs represent the CPM values that were greater than 20 in one group while less than 20 in the other group. Similarly, for other diagrams, e.g., the number of tRF-1s, of which the CPM in the sample or the average CPM in the group was not less than 20, can be calculated against the length of the sequence or tRNA isodecoders. Venn diagrams were plotted with the R VennDiagram package. Heatmaps were produced based on the differentially expressed tsRNAs with the R pheatmap package.

### The artificial synthesis of tsRNAs and injection in vivo

Modified tsRNAs were artificially synthesized by Beijing Huigene Biotechnology Corporation. PS modifications were performed in N−1 bases of the 5’ and first, second base of 3’ to prevent RNA degradation and improve the ability of RNAs to enter cells. Modification of 2’-OMe was performed in the whole RNA strand to increase affinity and avoid degradation. Cholesterol modification was added to the 3’ end to increase the ability of RNAs to enter cells. Each type of tsRNAs was mixed in equal amounts. A random code sequence was used as a negative control. In addition, the 68 screened tsRNAs with or without modifications were synthesized by the Beijing Huigene Biotechnology Corporation. The negative control of synthesized tsRNAs was 5’-UUCUCCGAACGUGUCACGUTT-3’. Other sequences are presented in Supplementary Table S1.

We then diluted 50 μg mixed tRF-1s (tRF-Gln-CTG-026, tRF-Met-CAT-049, or control RNAs can be used alternatively) with an appropriate amount of endotoxin-free pure water to a final concentration of 1 μg/μL and added 50 μL 10% glucose solution (w/v) to yield a final glucose concentration 5%; the final volume was 100 μL. The solutions were adequately mixed. We then mixed 25 μL transfection reagent in vivo (Entranster in Vivo, 18668-11-1) with 50 μL of 10% glucose solution and added 25 μL pure water to make a final glucose concentration of 5%. The final volume was 100 μL and the solution was mixed well. The diluted transfection reagent was added to the diluted RNA solution at room temperature and immediately shaken and mixed thoroughly. The solution was kept at room temperature for 15 min and was intravenously injected.

### Determine the injection condition using tRF-Met-049

First, the mice received an intravenous injection of tRF-Met-049 solution (10 μL/g). The mice in the control group were intravenously injected with the control solution (10 μL/g). The Blank group marked as D0 did not receive any RNA. At days 1, 2, 3, and 4 after injection, the mice were sacrificed, and liver tissues were collected to perform a qRT-PCR assay to determine the expression of injected tsRNAs to determine the time at which tsRNAs should be injected.

### Partial hepatectomy (PH)

A two-thirds partial hepatectomy was performed as reported before.^39^ Briefly, an incision was made along the midline of the abdomen to expose the liver. The falciform, hepatogastric, and part of the left coronary ligaments were cut. The hepatic artery, and portal vein branches innervate left lateral and middle hepatic lobes were ligated with a 7–0 suture. The roots of the left lateral, left middle, and right middle lobes were ligated with 4–0 sutures, and then excised. The abdominal cavity was cleaned, and the abdomen was closed. Finally, samples were collected for further study, and the Ki67 staining results were shown with Vectra Polaris (Akoya).

### RNA pull-down

The RNA pull-down assay was performed according to instructions (Pierce Magnetic RNA-Protein Pull-Down Kit, 20164). In brief, artificially synthesized tRF-Gln-CTG-026 was biotinylated. Streptavidin Magnetic Beads (NEB, S1420S) were pre-washed, and the cell lysate was prepared; the protein concentration was required to be more than 2 mg/mL. The Streptavidin Magnetic Beads were washed with 50 μL 20 mM Tris (v/v), and 50 μL 1 × RNA Capture Buffer was added and mixed well. After 50 pM labeled RNA was added, the solution was kept at room temperature for 1 h. Then, 20 mM Tris (pH 7.5) (v/v) was added to wash the beads. Protein–RNA Binding Buffer (1×) was added into the beads and mixed well. RNA–protein mix was added to the beads for 3 h at 4 °C. Finally, the samples were washed and eluted.

### Proteome detection

This experiment was performed by Novogene Co. Ltd. RNA pull-down samples were injected into an ultrafiltration system and were centrifuged at 14,000 × *g* for 15 min. The protein was buffered with DB buffer (8 M Urea, 100 mM TEAB, pH 8.5). 10 mM DTT was added into collected supernatant and kept for 1 h at 56 °C. Then, excess iodoacetamide was added into the supernatant and kept for 1 h in the dark at room temperature. After quantifying the protein concentration with the Bradford kit as specified by the user’s manual, SDS-PAGE gel electrophoresis was performed to test the protein quality. Then, 120 μg of protein, along with DB lysis buffer (8 M Urea, 100 mM TEAB, pH 8.5), trypsin, and 100 mM TEAB buffer, was dissolved to make 100 μL of solution. The solution was kept for 4 h at 37 °C. Trypsin and CaCl_2_ were added to digest the sample overnight. Formic acid was added to make the solution pH < 3. The samples were centrifuged at 12,000 × *g* for 5 min at room temperature, and the pellets were discarded. The supernatant was loaded onto a C18 desalting column, washed with 0.1% formic acid and 3% acetonitrile three times, and eluted with 0.1% formic acid and 70% acetonitrile. The eluents were collected and lyophilized. Mobile phase A and B were prepared according to the manual of Novogene. The lyophilized powder was dissolved in 10 μL A solution. The samples in solution A were centrifuged at 14,000 × *g*, 4 °C for 20 min, and the pellets were discarded. Novogene used several devices to perform the LC-MS/MS analysis: EASY-nLC 1200 UHPLC system (Thermo Fisher, LC40); Orbitrap Exploris 480 matched with FAIMS (Thermo Fisher), home-made C18 Nano-Trap column (4.5 cm × 75 μm, 3 μm); home-made analytical column (25 cm × 150 μm, 1.9 μm); Nanospray Flex (ESI).

### Proteome analysis

The software Proteome Discoverer 2.4 (PD 2.4, Thermo) was used to search the spectral results against protein databases. The software parameters used were: mass tolerance of precursor ion, 10 ppm; mass tolerance of product ion, 0.02 Da; fixed modifications, carbamidomethyl; dynamic modification, oxidation of methionine (M); N-Terminal: acetylation Met-loss and Met-loss + acetylation; maximum 2 mis-cleavage sites allowed. The results were filters through the following conditions: identified Peptide Spectrum Matches (PSMs) are >99% credibility and > = 1 unique peptide in identified protein; FDR verification, discard >1% result; the *t*-test was used for statistical analysis of the results.

### RNA immunoprecipitation

RIP was performed in line with the manufacturer’s instructions (Magna RIP RNA-Binding Protein Immunoprecipitation Kit, 17–700). Briefly, HL-7702 and AML12 cells were first transfected with Flag-TSR1 and tRF-Gln-CTG-026, harvested, resuspended in an equal volume of RIP lysis buffer, and kept on ice for 5 min. Then, 200 μL of lysate was frozen at −80 °C. An appropriate amount of Flag beads (ab270704) was washed once with 0.5 mL RIP wash buffer and then resuspend in RIP wash buffer. RIP Immunoprecipitation Buffer was prepared (35 μL 0.5 M EDTA, 5 μL RNase inhibitor, 860 μL RIP wash buffer) and added to the beads. RIP lysate was thawed and centrifuged at 12,000 × *g* for 10 min at 4 °C. Then, 100 μL of the supernatant was removed added to the beads; 10 μL was taken as the input and stored at −80 °C. The expression of RIP protein and RNA from input samples was detected by western blotting. RIP samples were placed on a rotator at 4 °C for 3 h. The sample was centrifuged and the supernatant was discarded. After one wash with 0.5 mL RIP wash buffer and six washes with ice-cold RIP wash buffer, proteinase K solution was added, and the protein was digested at 55 °C. After a further wash with RNA wash buffer, RNA was extracted and qRT-PCR analysis was performed.

### Detection of global protein synthesis in vitro and in vivo

For the detection of GPS in vitro, we used a protein detection kit and performed experiments according to kit instructions (Protein Synthesis Assay Kit, ab239725). Briefly, the cells were seeded and stimulated with H_2_O_2_. The medium was changed to Protein Label (1×) and the cells were culture at 37 °C for 2 h. The medium was removed, the cells were washed once with 100 μL PBS, and the supernatant was discarded. Then, 100 μL of fixative solution was added and the cells were kept for 15 min at room temperature in the dark. The fixative solution was removed and the cells were washed once with 200 μL of 1× wash buffer. Then, 100 μL of 1× permeabilization buffer was added, incubated for 10 min at room temperature, and the supernatant was discarded. The reaction cocktail was prepared, and 100 μL of 1× reaction cocktail was added and incubated at room temperature for 30 min in the dark. After incubation, the reaction cocktail was discarded and the cells were washed twice with 200 μL wash buffer. The wash buffer was discarded and the cells were resuspended in 100 μL PBS. Then, 1× DNA Stain was added for DNA staining and the sample was protected from light for 20 min at room temperature. For GPS detection in vivo, 25 mg/kg puromycin dihydrogen salt was injected i.p., with a total injection volume of 100 μL, 1 h before the mice were killed. Western blotting showed the GPS results (Puro antibody, MABE343) (Goodman and Hornberger, 2013). Silver staining was used as an internal control.

### Statistical analysis

Statistical analyses (mean ± standard deviation [SD]) were calculated by GraphPad Prism 8 or R 3.5.0 or Excel 2019 unless specifically indicated in the figure legends. Two-tailed Student’s *t*-tests were used to test the statistical significance between samples. Analysis of variance (ANOVA) was used to analyze the significance between more than two groups. Data analysis was performed using GraphPad Prism 8. Diagrams were prepared using Excel software. Image inversion, cropping, and indicated area marking were performed using Photoshop. The cell counts and indicated areas of the images were calculated using ImageJ software. For all the data in this article, *n.s*. represents *P* > 0.05; *P < 0.05; **P < 0.01.

## Supplementary information

Supplementary Information

Supplementary Information, Table S2

## Data Availability

tsRNA sequencing profiling data and raw data have been uploaded to Gene Expression Omnibus (GEO) with the following accession numbers: GSE144698 according to the journal publication policy. The mass spectrometry and proteomics data have been deposited to the ProteomeXchange Consortium (http://proteomecentral.proteomexchange.org) via the iProX partner repository^40^ with the dataset identifier PXD033097. This study did not generate any code. Further information and requests for resources and reagents should be directed to and will be fulfilled by the lead contact, Qi ZHOU (zhouqi@ioz.ac.cn).
