# Supplementary material for: tRF-Gln-CTG-026 ameliorates liver injury by alleviating global protein synthesis
Source: Signal Transduct Target Ther. 2023 Apr 3;8:144. doi: 10.1038/s41392-023-01351-5 (PMC10073094; doi:10.1038/s41392-023-01351-5)
Supplement: Supplementary file 1 — Supplementary Information [file 41392_2023_1351_MOESM1_ESM.docx]

Supplementary Materials for

tRF-Gln-CTG-026 ameliorates liver injury through alleviating global protein synthesis

Sunyang Ying, Pengcheng Li, Jiaqiang Wang, Kaiqiong Chen, Yu Zou, Moyu Dai, Kai Xu, Guihai Feng, Changjian Zhang, Haiping Jiang, Wei Li, Ying Zhang*, Qi Zhou*

*Correspondence to: Qi Zhou (zhouqi@ioz.ac.cn), Ying Zhang (yingzhang@ioz.ac.cn)

**This PDF file includes:**

Figures. S1 to S6

Tables S1 to S3

**Other Supplementary Materials for this manuscript include the following:**

Supplementary Table S2: TIC_Raw and Normalized Peak Information


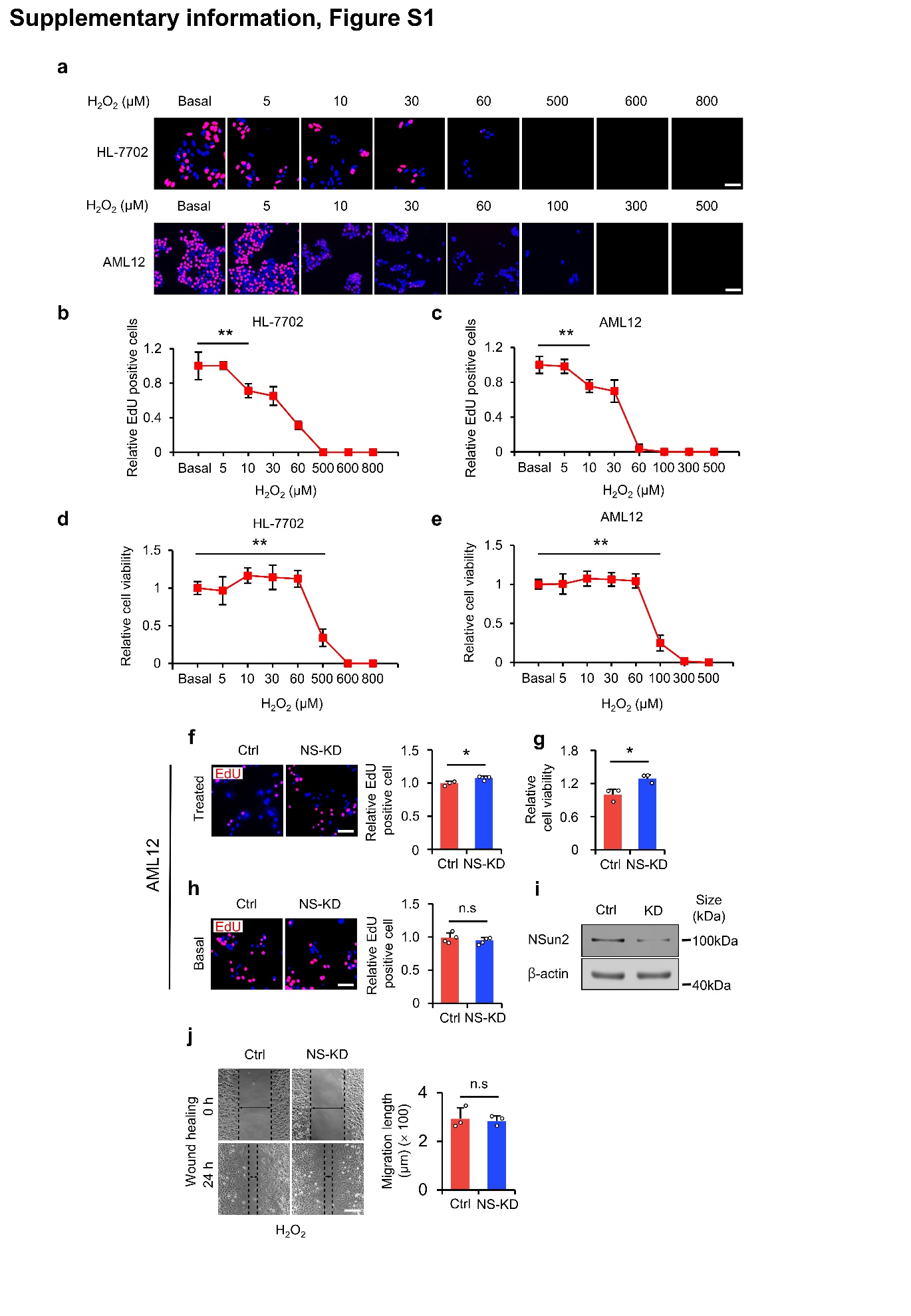


Fig. S1. NS-KD ameliorates injury in AML12 cells.

(**a**) The effect of H_2_O_2_ dose on HL-7702 and AML12 cell proliferation. Representative images of EdU staining are shown. Basal: no H_2_O_2_. Scale bar, 100 μm. (**b**) The statistical results of HL-7702 cell proliferation under H_2_O_2_ stress in Supplementary Fig. S1a. EdU-positive cells were analyzed relative to the Basal. (**c**) The statistical results of AML12 cell proliferation under H_2_O_2_ stress in Supplementary Fig. S1a. EdU-positive cells were analyzed relative to the Basal. (**d**) Relative HL-7702 cell viability (CCK-8 assay) under different H_2_O_2_ dose was assessed by detecting the OD450 value. (**e**) Relative AML12 cell viability (CCK-8 assay) under different H_2_O_2_ dose was assessed by detecting the OD450 value. (**f**) The effect of NS-KD on AML12 cell proliferation under H_2_O_2_ stress. Treated: H_2_O_2_. Left panel: representative images of EdU staining are shown. Right panel: statistical results. Scale bar, 100 μm. EdU-positive cells were analyzed relative to the control. *n* = 3 replicates. (**g**) Relative AML12 cell viability (CCK-8 assay) was assessed by detecting the OD450 value. *n* = 3 replicates. (**h**) Detection of transfected AML12 proliferation in the absence of stress damage. Basal: no H_2_O_2._ Left panel: representative images of EdU staining are shown. Right panel: statistical results. Scale bar, 100 μm. EdU-positive cells were analyzed relative to the control. *n* = 4 replicates. (**i**) NSun2 expression was measured to verify the knockdown effect of NSun2 by western blotting for Supplementary Fig. S1a-c. (**j**) Cell migration distances in the wound healing assay at 0 h and 24 h (left panel) and quantified (right panel). Scale bar, 200 μm. Data are representative of three independent experiments. *NSun2* siRNA: NS-KD; Ctrl siRNA: Ctrl. All data are expressed as the mean ± SD. **P* < 0.05, ***P* < 0.01, *n.s.* no significance, Student’s *t*-test.


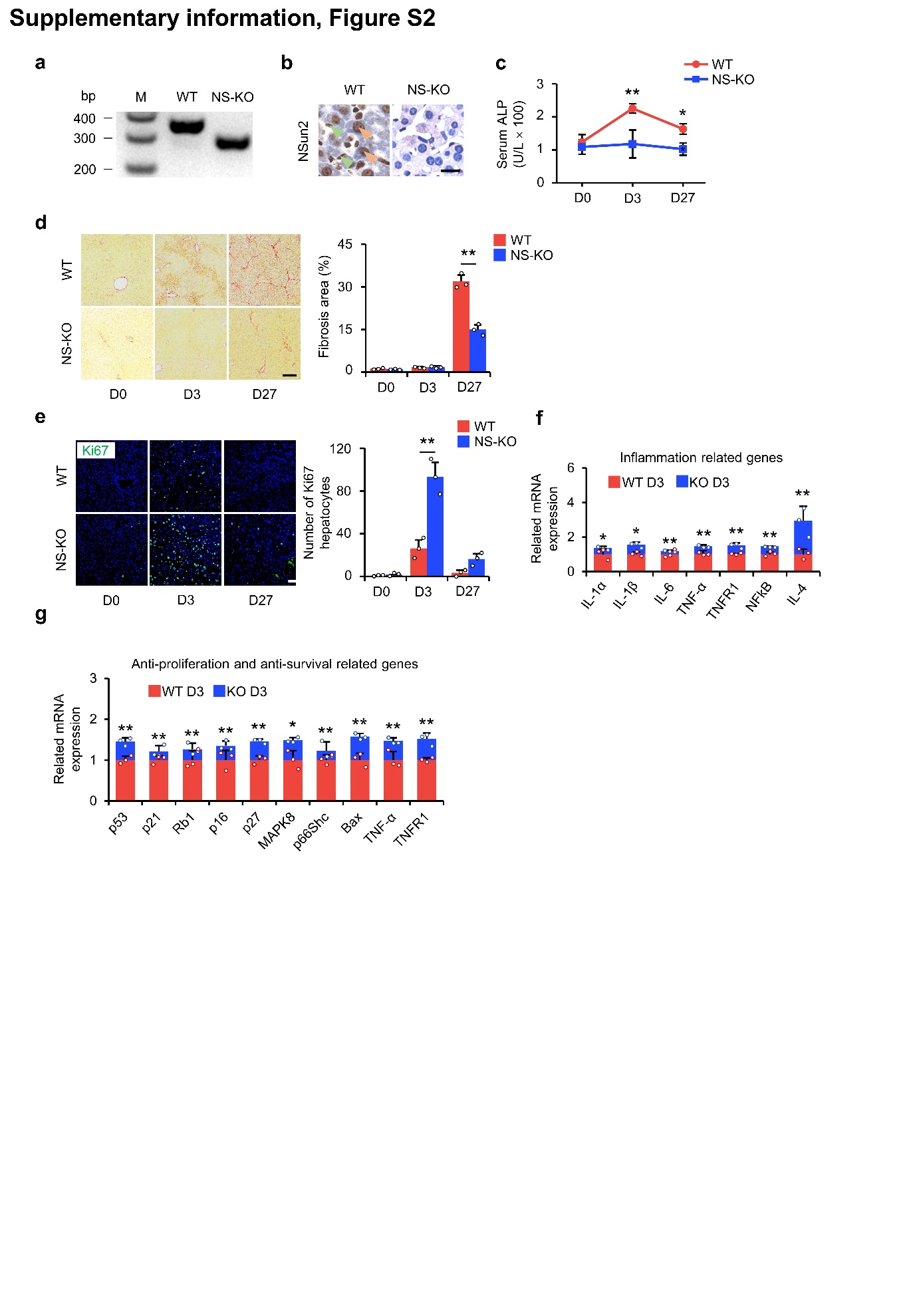


Fig. S2. NS-KO ameliorates liver fibrosis, inflammation, proliferation, and survival *in vivo.*

(**a**) Genotyping analysis of WT and NS-KO mice by DNA gel electrophoresis. (**b**) immunohistochemistry showing NSun2 location and effect of NS-KO in liver sections. The brown cells represent NSun2-positive cells. The larger nuclei represent hepatocytes (orange arrowhead), and other smaller nuclei represent non-parenchymal cells (green arrowhead). Scale bar, 50 μm. (**c**) Mouse serum ALP concentration was assessed to manifest the degree of liver injury after repeated CCl_4_ injection, *n* = 4 mice. (**d**) Mouse liver fibrosis is induced by repeated injections of CCl_4_. Sirius red staining was used to detect collagen (red area) in the injured liver at different time points, and the percentage of the fibrotic area was quantified. Scale bars, 200 μm; *n* = 3 mice. (**e**) Immunofluorescence of Ki67 staining in liver sections. WT or NS-KO mice were injected with CCl_4_. The liver was resected and made into sections at the indicated time points. Left panel: representative images of Ki67-positive hepatocytes (green stained large nuclei). Right panel: Ki67-positive cells were quantified. Scale bar, 100 μm. *n* = 3 mice. (**f**) Inflammation-related gene expression was quantified by qRT-PCR after liver injury. *n* = 3 mice. (**g**) Anti-proliferation and injury-related gene expression was quantified by qRT-PCR after liver injury. *n* = 3 mice. For data in this figure, representative images are shown. All data are presented as the mean ± SD. **P* < 0.05, ** *P* < 0.01, Student’s *t*-test.


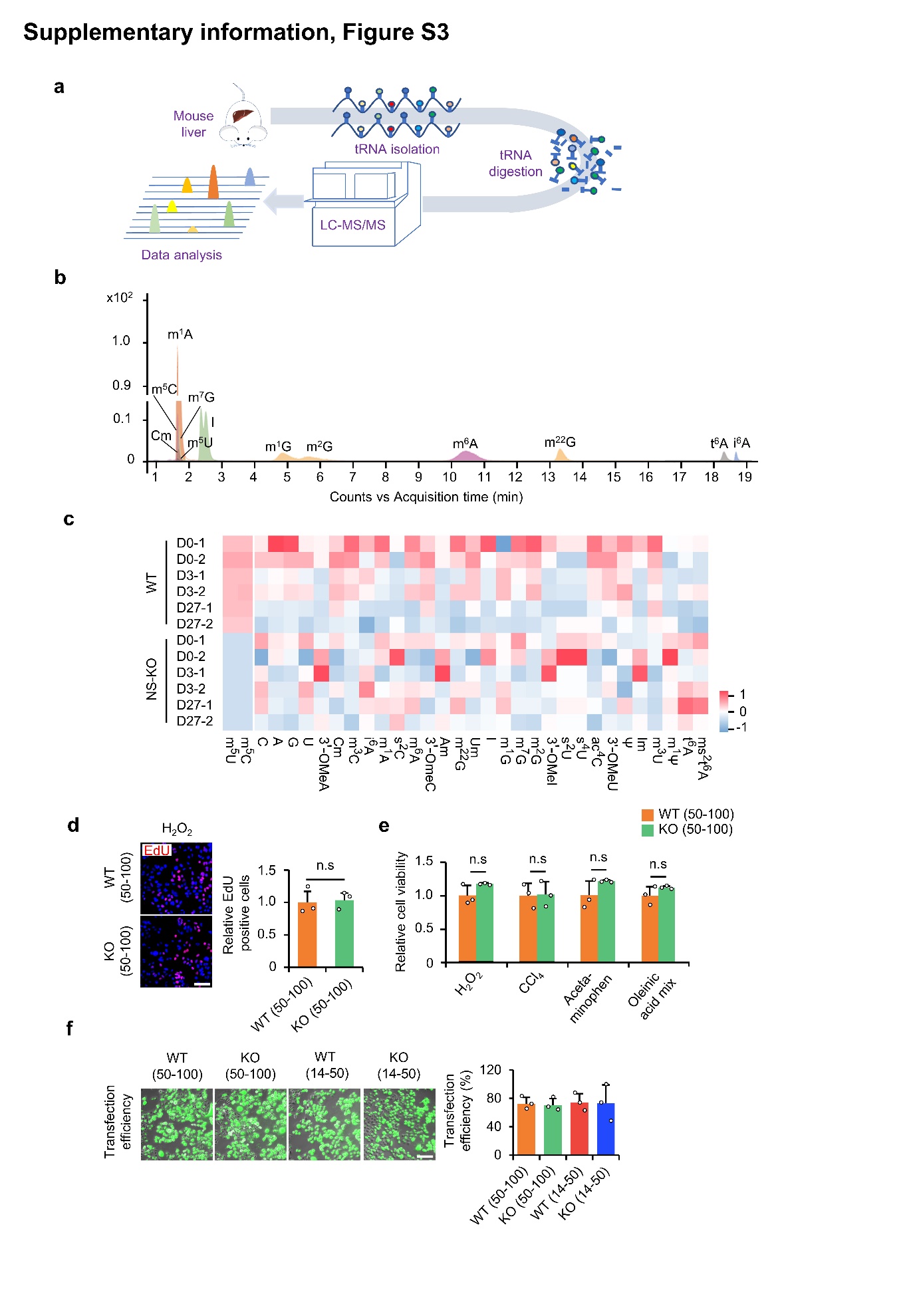


Fig. S3. The profile of tRNA modifications after injury and the roles of 50–100 nt RNAs from NS-KO in cell injury.

(**a**) Schematic diagram of experimental procedures for detecting and quantifying multiple tRNA modifications in the mouse liver. Briefly, total RNA from each sample was quantified using a NanoDrop ND-1000 instrument, and ~5 μg total RNA was used to isolate tRNA by the Urea-PAGE method. Purified tRNA was digested to single dephosphorylated nucleosides by enzyme mix. LC-MS analysis was performed on Agilent 6460 QQQ mass spectrometer with an Agilent 1260 HPLC system using multi-reaction monitoring (MRM) detection mode. LS-MS data were acquired using Agilent Qualitative Analysis software. MRM peaks of each modified nucleoside were extracted and normalized to the quantity of tRNA purified. (**b**) Representative total ion current (TIC) results of nucleosides. This image is provided in Excel format for each sample in the Supplemental information (SupplementaryTable S2). (**c**) Heatmap showing the relative abundance of 41 types of tRNA modifications in WT and NS-KO mice treated with CCl_4_. 32 of the 41 modifications are available. They are m^5^U, m^5^C, C, A, G, U, 3'-OMeA, Cm, m^3^C, i^6^A, m^1^A, s^2^C, m^6^A, 3'-OmeC, Am, m^22^G, Um, I, m^1^G, m^7^G, m^2^G, 3'-OMeI, s^2^U, s^4^U, ac^4^C, 3'-OMeU, Ψ, Im, m^3^U, m^1^Ψ, t^6^A, ms^2^t^6^A. 9 of them are N.D. They are m^5^Cm, m^2,2,7^G, ac^4^Cm, 5'-OMeT, Gm, m^5^s^2^U, mo^5^U, hm^5^C, m^5^Um. The horizontal axis represents the type of tRNA modification. The vertical axis represents the time point when WT and NS-KO mice were treated with CCl_4_. -1 and -2 indicate a repeated sample. For the specific data, the raw and normalized peak information datasheet of detected modified nucleosides are provided in the same excel datasheet as table S2. Nucleoside, the name of normal or modified nucleoside detected; Symbol, an abbreviation of nucleoside detected; Raw Peak Area, Peak area extracted from LC-MS data; Normalized Peak Area, Peak area normalized to the quantity of purified tRNA for each sample. N.D., Not Detected, nucleoside with a signal-to-noise ratio below 10. The data shown are the results of two independent experiments. (**d**) Cell proliferation after transfection of 50–100 nt RNAs was assessed after injury using the EdU incorporation assay (left panel); scale bar, 100 μm. Cell proliferation was quantified (right panel). *n* = 3 replicates. (**e**) CCK-8 assay showing the survival of isolated fragments. Relative cell viability after transfection of 50–100 nt RNAs was assessed by detecting the OD450 value. *n* = 4 replicates. (**f**) Transfection efficiency of RNA fragments isolated from WT and *NSun2* KO mice. Representative green fluorescence-positive cells are shown (left panel), scale bar, 100 μm. Green fluorescence-positive cells were quantified (right panel). Six representative fields for each sample were selected to assess the transfection efficiency. Images are representative of three independent experiments. All data are expressed as the mean ± SD. **P* < 0.05, ***P* < 0.01, *n.s.* no significance, Student’s *t*-test.


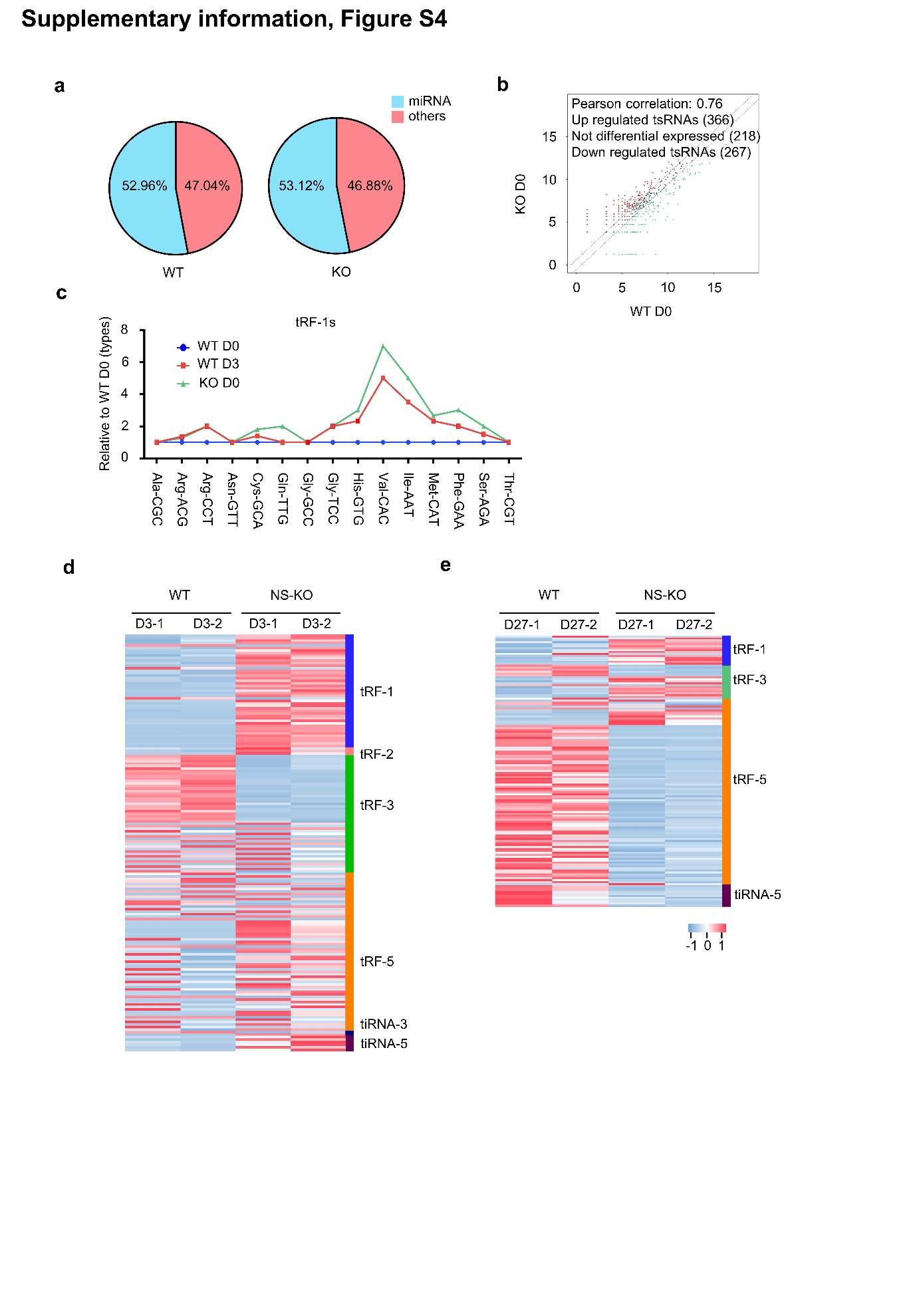


Fig. S4. The landscape of tsRNAs from WT and NS-KO liver at different time points.

(**a**) The distribution of miRNAs and other RNAs after NS-KO. (**b**) The scatter plot of differentially expressed tsRNAs between WT D0 and KO D0. tsRNAs above the top line (red dots, upregulation) or below the bottom line (green dots, downregulation) indicate more than 1.5-fold change between the two compared groups. Gray dots indicate non-differentially expressed tsRNAs. (**c**) The number of tRF-1s against tRNA isodecoders. tRNA isodecoders share the same anticodon but have differences in their body sequence. The X-axis represents different tRNA isodecoders. The Y-axis shows the number of all tRF-1s against tRNA isodecoders relative to WT D0. The lines with different colors represent multiple samples. tsRNAs are separated into subtypes by their sites and length. The group line chart showing the number distribution for each subtype of tsRNAs. (**d** and **e**) Related to Fig. 4e, the differentially expressed tsRNAs of other compared groups. All data in this figure were acquired by tsRNA-seq; for each group, two duplicates were performed.


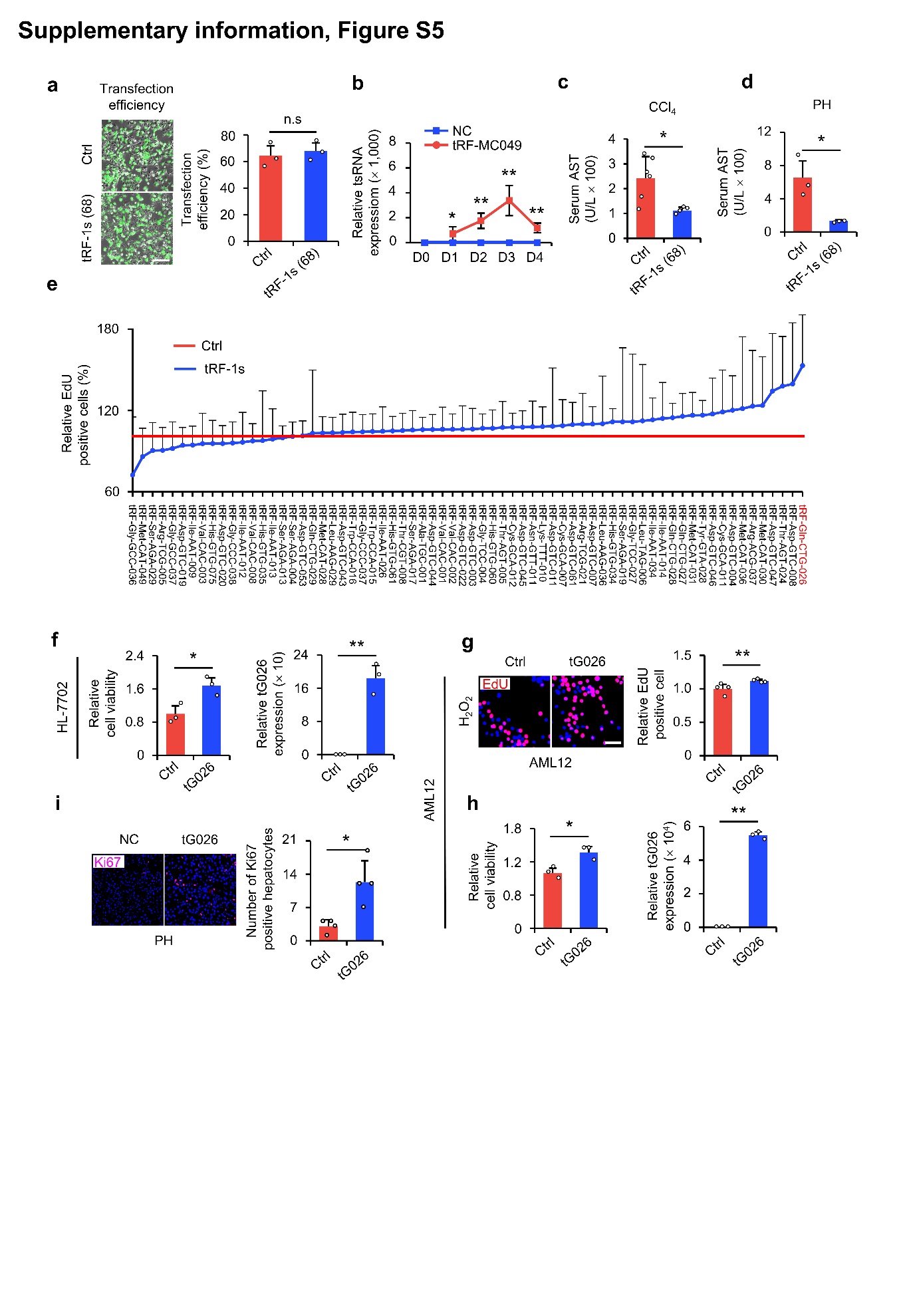


Fig. S5. The screening of NS-KO-derived tRF-1s that can improve liver injury.

(**a**) Transfection efficiency of synthetic tRF-1s. After mixing tRF-1s and Block-iT oligos with the green fluorescence, the mixtures were transfected into HL-7702 cells for indirect detection of the transfection efficiency. Green fluorescence-positive cells can be considered as cells transfected with synthetic tRF-1s. Representative green fluorescence-positive cells are shown (left panel) and quantified (right panel). Six representative fields from each sample were selected to assess the transfection efficiency. Scale bar, 100 μm. The experiments above were performed three times. (**b**) qRT-PCR determined the retention time of injected tRF-MC049 (tRF-Met-CAT-049) in the mouse liver. Random code sequence (NC) as a negative control. D0 represents no injection. D1, D2, D3, and D4 represent 0, 1, 2, and 3 days after small RNAs injection. (**c**) Related to Fig. 5g, another detected liver function index—AST for CCl_4_-induced liver injury. Control mice, *n* = 6; tRF-1s mice, *n* = 4. (**d**) Related to Fig. 5i, another detected liver function index—AST for partial hepatectomy (PH) liver injury model. *n* = 3 mice. (**e**) EdU staining showing relative HL-7702 proliferation under H_2_O_2_ stress for each screened tRF-1. The red font is the optimal tRF-1—tG026. (**f**) Left panel: relative HL-7702 viability (CCK-8 assay) was assessed by detecting the OD450 value. Right panel: qRT-PCR showing the identification of tG026 expression. *n* = 3 replicates. (**g**) AML12 cells were transfected with artificial synthetical tG026 or a random code sequence as negative control. EdU-positive cells were analyzed relative to the control. Representative images (left panel) and statistical results (right panel) are shown. Scale bar, 100 μm. *n* = 4 replicates. (**h**) Left panel: relative AML12 viability (CCK-8 assay) was assessed by detecting the OD450 value. Right panel: qRT-PCR showing the identification of tG026 expression. *n* = 3 replicates. (**i**) After partial hepatectomy (PH), the different proliferation between Ctrl and tG026-injected mice was compared using EdU staining. Representative images (left panel) and statistical results (right panel) are shown. Scale bar, 50 μm. *n* = 4 mice. All data are expressed as the mean ± SD. **P* < 0.05, ***P* < 0.01, *n.s.* no significance, Student’s *t*-test.


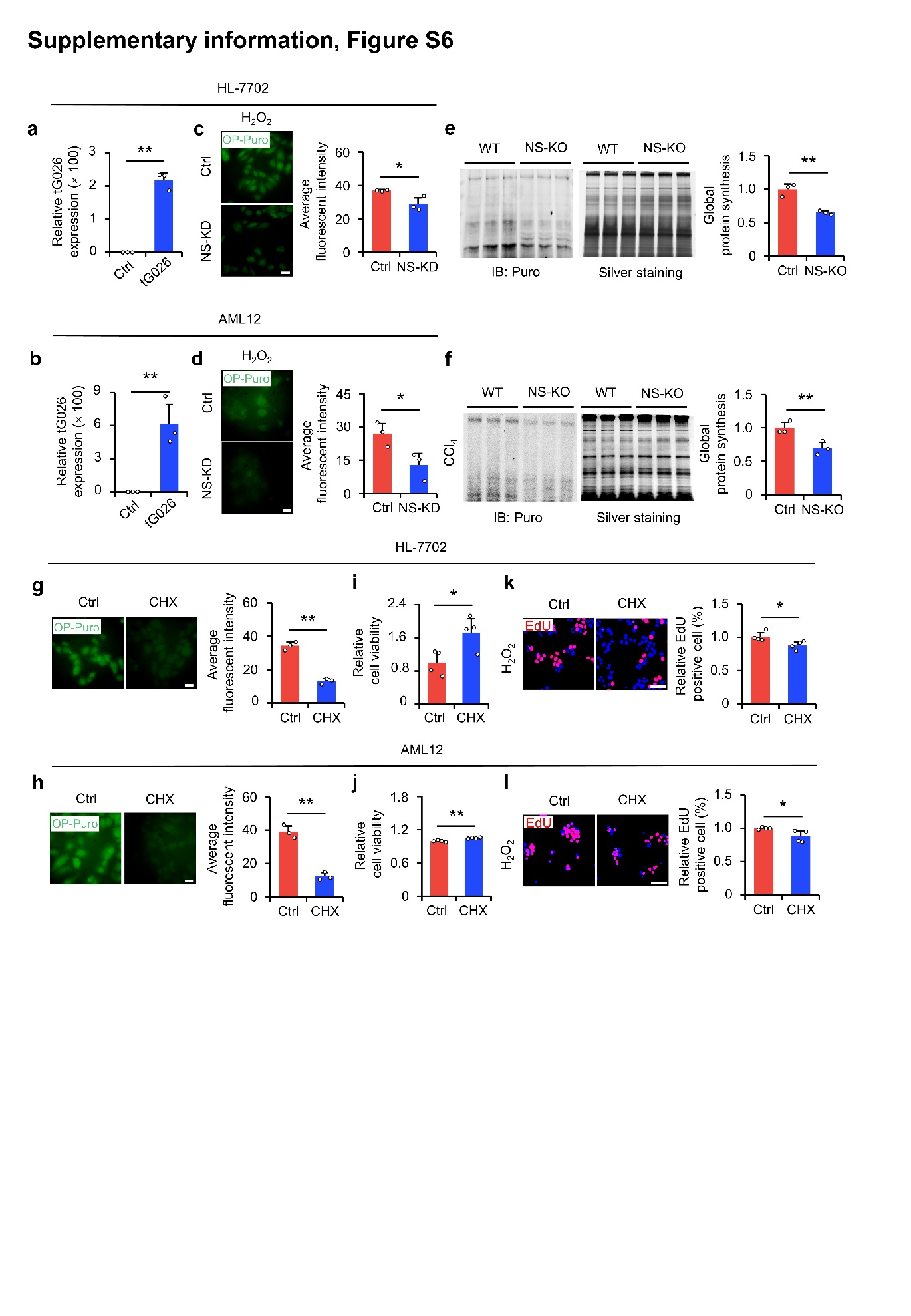


Fig. S6. The loss of *NSun2* suppresses the global protein synthesis in the presence or absence of stress and the effect of CHX on cell injury.

(**a**) The identification of tG026 expression in Fig. 6e. *n* = 3 replicates. (**b**) The identification of tG026 expression in Fig. 6f. *n* = 3 replicates. (**c** and **d**) Global protein synthesis under H_2_O_2_ stress in HL-7702 cells (c) and AML12 cells (d) was assayed by OP-Puro incorporation. Results showing the variation after NS-KD. Representative images (left panel) and statistical results (right panel) are shown. Scale bar, 50 μm. *n* = 3 replicates. (**e** and **f**) SUnSET showing GPS *in vivo* with (e) or without damage stress (f) after NS-KO. Left panel: the quantity of Puro. Middle panel: total protein quantity as an internal control. Right panel: grey intensity statistical results. *n* = 3 mice. (**g** and **h**) Global protein synthesis in HL-7702 cells (g) and AML12 cells (h) was assayed by OP-Puro incorporation. Results showing the variation after being treated by CHX. Representative images (left panel) and statistical results (right panel) are shown. Scale bar, 50 μm. *n* = 3 replicates. (**i** and **j**) Relative cell viability (CCK-8 assay) in HL-7702 (i) and AML12 (j) was assessed by detecting the OD450 value. *n* = 4 replicates. (**k** and **l**) The cell proliferation under H_2_O_2_ stress after GPS was inhibited by CHX. HL-7702 (k) or AML12 (l) were treated with CHX. Representative images (left panel) and statistical results are (right panel) are shown. Scale bar, 100 μm. *n* = 4 replicates. All data are expressed as the mean ± SD. **P* < 0.05, ***P* < 0.01, Student’s *t*-test.

Table S1. NS-KO-derived 68 tRF-1s sequences

| Screened standard | Name | Sequence (5' - 3') |
| --- | --- | --- |
| WT D0  vs  KO D0  log2 > 2  p < 0.05 | tRF-Ser-AGA-017 | TCTAGGATTTCTTTT |
|  | tRF-Trp-CCA-015 | TCTGTGTTCTTTATTT |
|  | tRF-Trp-CCA-016 | TCTGTGTTCTTTATTTT |
|  | tRF-Gly-TCC-027 | TAAGGTTGTCTTTT |
|  | tRF-Ile-AAT-014 | GTGTGCTTATCTTT |
|  | tRF-Met-CAT-028 | TCGGAGGCTTTGTTTTTA |
|  | tRF-Met-CAT-031 | TCTGGACTTTTTTTT |
|  | tRF-Val-CAC-008 | ATGACTCTTTTTTT |
|  | tRF-Gly-TCC-004 | ACGGCAAACCTTTT |
| WT D3  vs  KO D3  log2 > 2  p < 0.05 | tRF-Met-CAT-030 | TCTGGACTTTTTTT |
|  | tRF-Ser-AGA-029 | TCATGGTCTGTTTT |
|  | tRF-Asp-GTC-011 | ATACAGTAGCTTTTT |
|  | tRF-Asp-GTC-007 | AGTGATCTCTATTTT |
|  | tRF-Asp-GTC-008 | AGTGATCTCTATTTTT |
|  | tRF-Ile-AAT-012 | GTGGTAGATTCTTT |
|  | tRF-Gln-CTG-028 | TTGGTGTCAGGCTAGTTTTCTT |
|  | tRF-Ile-AAT-013 | GTGGTAGATTCTTTT |
|  | tRF-Asp-GTC-046 | TCTGGACACATGTGGCTTTTTG |
|  | tRF-Ile-AAT-009 | GTAAACTGCTTAGATTT |
|  | tRF-Arg-TCG-005 | AGCTAGTGTTCTTTT |
|  | tRF-Gly-GCC-037 | GAGTCCAGTCCCTTTT |
|  | tRF-Ala-TGC-001 | AACGGCTACCGCCTACCTTT |
|  | tRF-Asp-GTC-003 | ACGTAGCGTCCTTTT |
|  | tRF-His-GTG-061 | GTATTGTATTCTGTGCCAGCATCTTTT |
|  | tRF-Asp-GTC-020 | GCTAACAGTGGTTTT |
|  | tRF-His-GTG-060 | GTATTGTATTCTGTGCCAGCATCTTT |
|  | tRF-Val-CAC-002 | ACACTTGTCAGTTTCTTT |
|  | tRF-Asp-GTC-047 | TCTGGACACATGTGGCTTTTTGTTT |
|  | tRF-Asn-GTT-011 | GTCTCGGCCTTTTTTTT |
|  | tRF-Val-CAC-003 | ACACTTGTCAGTTTCTTTT |
|  | tRF-His-GTG-075 | GTATTGTATTCTGTGCCAGCATCTT |
|  | tRF-Gly-CCC-037 | GTACCTCTCTGTTT |
|  | tRF-Asp-GTC-019 | GCGTAGCGTCCTTTT |
|  | tRF-Asp-GTC-053 | ACGTAGCGTCCTTT |
|  | tRF-Gln-CTG-027 | TTGGTGTCAGGCTAGTTTT |
|  | tRF-Gln-CTG-026 | TTGGTGTCAGGCTAGTTT |
|  | tRF-His-GTG-034 | TTATCCTCTGGTCACTTTT |
|  | tRF-Gln-CTG-029 | TTGGTGTCAGGCTAGTTTTCTTT |
| **Continued** |  |  |
| Screened standard | Name | Sequence (5' - 3') |
| WT D3  vs  KO D3  log2 > 2  p < 0.05 | tRF-Asp-GTC-061 | GCGTAGCGTCCTTTTT |
|  | tRF-Asp-GTC-045 | TCTGGACACATGTGGCTTTTT |
|  | tRF-Asp-GTC-043 | TCTGGACACATGTGGCTTT |
|  | tRF-Asp-GTC-044 | TCTGGACACATGTGGCTTTT |
|  | tRF-Gly-GCC-036 | GAGTCCAGTCCCTTT |
|  | tRF-Val-CAC-001 | ACACTTGTCAGTTTCTT |
|  | tRF-His-GTG-035 | TTATCCTCTGGTCACTTTTT |
|  | tRF-Lys-TTT-010 | GAAAGGTGATCGTTT |
|  | tRF-Gly-CCC-038 | GTACCTCTCTGTTTT |
| WT D0  vs  WT D3  log2 > 2 | tRF-Tyr-GTA-028 | AAGAGCTTGCTATTTT |
|  | tRF-Thr-CGT-008 | GCGTTTGGAAGAGATATTT |
|  | tRF-Thr-AGT-024 | TTGTGGTTTCTTATT |
|  | tRF-Thr-AGT-005 | GAGTGCTACCTTTT |
|  | tRF-Ser-AGA-019 | TCTAGGATTTCTTTTTT |
|  | tRF-Ser-AGA-013 | TCATGGTCTGTTTTA |
|  | tRF-Ser-AGA-004 | GAAGGGAATGTTTTTT |
|  | tRF-Met-CAT-049 | ATAAATCCTATCACCCTTGCT |
|  | tRF-Met-CAT-036 | ACCTCAGCTTTTATTTT |
|  | tRF-Leu-TAG-006 | ATCAATATTTTCACAACCTCAATCTTAT |
|  | tRF-Leu-AAG-036 | ACCTCAGCTTTTATTTT |
|  | tRF-Leu-AAG-029 | TCGGAGGCTTTGTTTTTAAAATTT |
|  | tRF-Ile-AAT-034 | GTAAACTGCTTAGATTTTA |
|  | tRF-Ile-AAT-026 | GTGGTAGATTCTTTTA |
|  | tRF-Cys-GCA-012 | CTACCGCCATTTTTT |
|  | tRF-Cys-GCA-011 | CTACCGCCATTTTT |
|  | tRF-Cys-GCA-007 | AGCAGGCTCACTCTTTTCTT |
|  | tRF-Asp-GTC-022 | GTACAATGATCATTTT |
|  | tRF-Asp-GTC-004 | ACGTAGCGTCCTTTTT |
|  | tRF-Arg-TCG-021 | AAATGTTTTCATTTGGATGGGATCTCTTT |
|  | tRF-Arg-ACG-037 | TTGGGACTGGTACTCCTTTATTTGT |

Negative Control: 5'-UUCUCCGAACGUGUCACGUTT-3'

Table S2. TIC_Raw and Normalized Peak Information (separate file)

Excel file containing detailed tRNA modification detection information.

Table S3. Primers and oligos used in this paper

| Used for | Name | Sequence (5' - 3') |
| --- | --- | --- |
| qRT-PCR | tRF-Met-CAT-049 Forward | GCCGGCATAAATCCTATCACC |
| qRT-PCR | tRF-Met-CAT-049 Reverse | ACTGCAGGGTCCGAGGTATT |
| qRT-PCR | tRF-Gln-CTG-026 Forward | CCGGCTTGGTGTCAGGC |
| qRT-PCR | tRF-Gln-CTG-026 Reverse | ACTGCAGGGTCCGAGGTATT |
| qRT-PCR | mouse NSun2 Forward | GGGACCAATTCATGGAGTCAC |
| qRT-PCR | mouse NSun2 Reverse | AACTTTGCCAACAACGGGGAC |
| qRT-PCR | human NSun2 Forward | CAAGCTGTTCGAGCACTACTAC |
| qRT-PCR | human NSun2 Reverse | CTCCCTGAGAGCGTCCATGA |
| qRT-PCR | human Dnmt2 Forward | GATGTCAACACTGTCG CTAATGA |
| qRT-PCR | human Dnmt2 Reverse | CCTTCAATCGTCTTGGCAAGT |
| qRT-PCR | human Mettl1 Forward | CCGACCCACATTTCAAGCG |
| qRT-PCR | human Mettl1 Reverse | TCCAGCACACGGTTATGGTA |
| qRT-PCR | mouse HGF Forward | ATGTGGGGGACCAAACTTCTG |
| qRT-PCR | mouse HGF Reverse | GGATGGCGACATGAAGCAG |
| qRT-PCR | mouse ID1 Forward | CCTAGCTGTTCGCTGAAGGC |
| qRT-PCR | mouse ID1 Reverse | CTCCGACAGACCAAGTACCAC |
| qRT-PCR | mouse c-Myc Forward | ATGCCCCTCAACGTGAACTTC |
| qRT-PCR | mouse c-Myc Reverse | CGCAACATAGGATGGAGAGCA |
| qRT-PCR | mouse Cyclin D1 Forward | GCGTACCCTGACACCAATCTC |
| qRT-PCR | mouse Cyclin D1 Reverse | CTCCTCTTCGCACTTCTGCTC |
| qRT-PCR | mouse CDK1 Forward | AGAAGGTACTTACGGTGTGGT |
| qRT-PCR | mouse CDK1 Reverse | GAGAGATTTCCCGAATTGCAGT |
| qRT-PCR | mouse c-Met Forward | CCCCAACTTCACGGCAGAAA |
| qRT-PCR | mouse c-Met Reverse | GTAGTTTGTGGCTCCGAGATAAA |
| qRT-PCR | mouse ERK Forward | GGTTGTTCCCAAATGCTGACT |
| qRT-PCR | mouse ERK Reverse | CAACTTCAATCCTCTTGTGAGGG |
| **Continued** |  |  |
| Used for | Name | Sequence (5' - 3') |
| qRT-PCR | mouse TGF-α Forward | CACTCTGGGTACGTGGGTG |
| qRT-PCR | mouse TGF-α Reverse | CACAGGTGATAATGAGGACAGC |
| qRT-PCR | mouse TNFR-1 Forward | CCGGGAGAAGAGGGATAGCTT |
| qRT-PCR | mouse TNFR-1 Reverse | TCGGACAGTCACTCACCAAGT |
| qRT-PCR | mouse IL-1α Forward | CGAAGACTACAGTTCTGCCATT |
| qRT-PCR | mouse IL-1α Reverse | GACGTTTCAGAGGTTCTCAGAG |
| qRT-PCR | mouse IL-1β Forward | GCAACTGTTCCTGAACTCAACT |
| qRT-PCR | mouse IL-1β Reverse | ATCTTTTGGGGTCCGTCAACT |
| qRT-PCR | mouse IL-6 Forward | TGGGGCTCTTCAAAAGCTCC |
| qRT-PCR | mouse IL-6 Reverse | AGGAACTATCACCGGATCTTCAA |
| qRT-PCR | mouse NFkB Forward | GGAGGCATGTTCGGTAGTGG |
| qRT-PCR | mouse NFkB Reverse | CCCTGCGTTGGATTTCGTG |
| qRT-PCR | mouse IL-4 Forward | GGTCTCAACCCCCAGCTAGT |
| qRT-PCR | mouse IL-4 Reverse | GCCGATGATCTCTCTCAAGTGAT |
| qRT-PCR | human 18S rRNA Forward | CGGCGACGACCCATTCGAAC |
| qRT-PCR | human 18S rRNA Reverse | GAATCGAACCCTGATTCCCCGTC |
| qRT-PCR | mouse 18S rRNA Forward | GCAATTATTCCCCATGAACG |
| qRT-PCR | mouse 18S rRNA Reverse | GGCCTCACTAAACCATCCAA |
| qRT-PCR | mouse β-actin Forward | GGCTGTATTCCCCTCCATCG |
| qRT-PCR | mouse β-actin Reverse | CCAGTTGGTAACAATGCCATGT |
| qRT-PCR | human β-actin Forward | CATGTACGTTGCTATCCAGGC |
| qRT-PCR | human β-actin Reverse | CTCCTTAATGTCACGCACGAT |
| qRT-PCR | mouse U6 Forward | CGCTTCGGCAGCACATATAC |
| qRT-PCR | mouse U6 Reverse | CACGAATTTGCGTGTCATCC |
| qRT-PCR | Human U6 Forward | TGCTTCGGCAGCACATATAC |
| qRT-PCR | Human U6 Reverse | TCACGAATTTGCGTGTCATC |
| **Continued** |  |  |
| Used for | Name | Sequence (5' - 3') |
| RT Primer | tRF-Met-CAT-049 | GTCGTATCGACTGCAGGGTCCGAGGTATTCGCAGTCGATACGACAGCAAG |
| RT Primer | tRF-Gln-CTG-026 | GTCGTATCGACTGCAGGGTCCGAGGTATTCGCAGTCGATACGACAAACTA |
| Genes Test | NS-KO genotyping Forward | AGCCTGAGGGCGAGGAAGAC |
| Genes Test | NS-KO genotyping Reverse | GGGATCCAGTGCCCTCTTCTGAT |
| NS-KO | sgNSun2-1 | GAACTCAAGATCGTGCCAGAGGG |
| NS-KO | sgNSun2-2 | GTACCCAGTGATTCTCAGTGTGG |
| Knockdown | human NSun2 siRNA1 | CCAGAUAGAUGUGGACGGCAGGAAA |
| Knockdown | human NSun2 siRNA2 | CGGCUGGCACAGGAGGGAAUAUAUA |
| Knockdown | human Mettl1 siRNA1 | GGGCTGGTGTATACCATAA |
| Knockdown | human Mettl1 siRNA2 | GATGGACTGGTCTGAGCTA |
| Knockdown | human Mettl1 siRNA3 | GGATGTGCACTCATTTCG A |
| Knockdown | human Dnmt2 siRNA1 | GCGATATGCTCTTCTGTTA |
| Knockdown | human Dnmt2 siRNA2 | CTGCGATATTTCACTCCTA |
| Knockdown | human Dnmt2 siRNA3 | GTAGTAGCTAAACTAATCA |
| Knockdown | mouse NSun2 siRNA1 | CCGTTTGGCACAGGAGGGAATATAT |
| Knockdown | mouse NSun2 siRNA2 | CCGTTGTTGGCAAAGTTCCATCAGT |
